# Electrochemical control of the single molecule conductance of a conjugated bis(pyrrolo)tetrathiafulvalene based molecular switch†
†This paper is dedicated to the memory of Prof. Thomas Wandlowski.
‡
‡Electronic supplementary information (ESI) available: Synthetic procedures, characterisation data, NMR spectra, MALDI mass spectra, details of CV measurements, details of break-junction measurements and data analysis, computational methods. See DOI: 10.1039/c7sc02037f



**DOI:** 10.1039/c7sc02037f

**Published:** 2017-06-23

**Authors:** Luke J. O'Driscoll, Joseph M. Hamill, Iain Grace, Bodil W. Nielsen, Eman Almutib, Yongchun Fu, Wenjing Hong, Colin J. Lambert, Jan O. Jeppesen

**Affiliations:** a Department of Physics , Chemistry and Pharmacy , University of Southern Denmark , Campusvej 55 , DK-5230 , Odense M , Denmark . Email: joj@sdu.dk; b Department of Chemistry and Biochemistry , University of Bern , Freiestrasse 3 , CH-3012 , Bern , Switzerland . Email: joseph.hamill@dcb.unibe.ch; c Department of Physics , Lancaster University , Lancaster , LA1 4YB , UK . Email: c.lambert@lancaster.ac.uk

## Abstract

As the field of unimolecular electronics develops, there is growing interest in the development of functionalised molecular wires, such as switches, which will allow for more complex molecular-scale circuits. To this end, a three redox state single molecule switch, **1**, based on bis(pyrrolo)tetrathiafulvalene (BPTTF) has been designed, synthesised and investigated using scanning tunnelling microscopy break junction (STM-BJ) studies and quantum transport calculations. Oxidising the BPTTF unit increases its conjugation, which was anticipated to increase the molecular conductance of **1**. By changing the redox state of **1** electrochemically it was possible to vary the single molecule conductance by more than an order of magnitude (from 10^–5.2^ *G*_0_ to 10^–3.8^ *G*_0_). Simulations afforded a qualitatively similar trend. An additional, higher conductance feature is present in most traces at junction sizes of around 2.0 nm – further extension affords the switchable lower conductance feature at junction sizes closer to the molecular length (*ca.* 3.0 nm). Analysis of the conductance traces shows that these two conductance features occur sequentially in nearly all junctions. This behaviour is attributed to an alternative initial junction conformation in which one or more of the BPTTF sulfur atoms acts as an anchoring group. This hypothesis is supported by a computational study of binding conformations and STM-BJ studies on a model compound, **2**, with only one thiol anchor. Our results indicate that the redox properties of BPTTF make it an excellent candidate for use in single molecule switches.

## Abstract 2

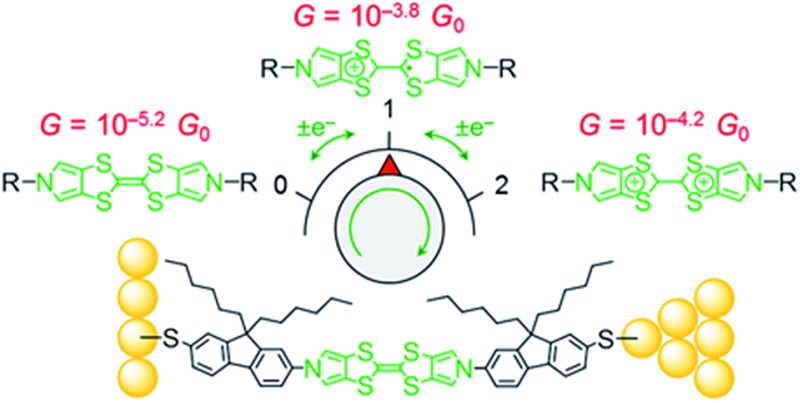
The single molecule conductance of a conjugated molecular wire is electrochemically switched upon oxidising or reducing a central bispyrrolotetrathiafulvalene unit.

## Introduction

The field of unimolecular electronics seeks to develop nanoscale, single molecule circuit components with the long-term goal of providing an alternative to silicon based technologies.^1^ The simplest such component is a molecular wire, which can be used to mediate electron transport between two electrodes. As methods of preparation and measurement of molecular wires have become better understood,^2–4^ interest has grown in incorporating additional functionality, for instance switching behaviour,^5^ into such systems. Conductance switching of functionalised molecular wires controlled by electrochemistry,^6–8^ light,^9^ pH^10^ and mechanical forces^11^ has been demonstrated previously. Irreversible chemically induced switching is also possible.^12^

The conductance of a single molecule can be measured using techniques such as the scanning tunnelling microscopy break junction^13^ (STM-BJ) and mechanically controlled break junction (MCBJ) methods.^2^ Both involve repeatedly forming and breaking a contact between two conductive elements in the presence of molecules that can be trapped in the resulting nanogap. Analysis of hundreds to thousands of conductance traces is then used to determine the most probable conductance of the molecule of interest. In STM-BJ experiments molecules are trapped between a metal substrate and an STM tip, whereas the MCBJ method uses the atomically sharp tips of two metallic wires. Gold is widely used to form the metallic contacts, although use of other metals such as palladium and platinum is possible.^3^ Each method can be more appropriate for a given experiment, for example, MCBJ can provide very fine mechanical control of the nanogap whereas STM-BJ is well suited to experiments requiring electrochemical gating.^3,14^ Recently, alternative nanogaps based on graphene have been investigated.^15,16^ These systems have the advantage of being amenable to electrostatic gating.

Molecular wires for single molecule conductance studies typically consist of a conjugated molecular backbone functionalised at either end with anchoring groups. By incorporating responsive groups into this general structure, molecular switches can be realised. Oligophenylene ethynylenes (OPEs) are archetypal backbones,^17^ which can be easily prepared using Sonogashira protocols. Other backbone architectures include those based on oligophenylene vinylenes (OPVs),^18^ oligothiophenes,^19^ oligoynes^20^ and larger π-systems such as fluorene derivatives.^21–23^ The choice of anchoring group is somewhat dependent on the substrate used in conductance studies. In the case of a gold substrate, a widely used anchoring group is the thiol moiety (typically protected by an acetyl group, which is cleaved *in situ*); other suitable anchoring moieties include amines, pyridine, cyano groups, thioethers and fullerenes.^2,20,24^ In contrast, planar aromatic anchors are favoured for graphene-based junctions.^16,25^

Redox-switching of the tetrathiafulvalene (TTF) unit has been extensively studied for applications in the field of supramolecular chemistry and has found use in sensors^26,27^ and molecular machines.^27,28^ TTF derivatives usually have three stable redox states (neutral state, radical cation and dication) which can be readily and reversibly accessed using electrochemical or chemical means.^29,30^ To exploit this switching behaviour, prior studies have incorporated the TTF unit into the backbone of molecular wires. Only limited examples exist of single molecule conductance studies on fully conjugated systems in which the conductance pathway runs through a TTF unit,^31,32^ and in these works only the conductance of the neutral state is reported. Studies of conductance switching have, to the best of our knowledge, been restricted to non-conjugated systems,^8^ which have an intrinsically low conductance, or extended TTF (exTTF) derivatives based on benzene^33^ (‘TTF cruciforms’) or anthraquinone.^34^ In these conjugated exTTF based wires the redox-active unit lies adjacent to the conductance pathway. In the former case this means that redox switching leads to a change from linear to cross conjugation, whereas in the latter the opposite process occurs.

**Chart 1:**
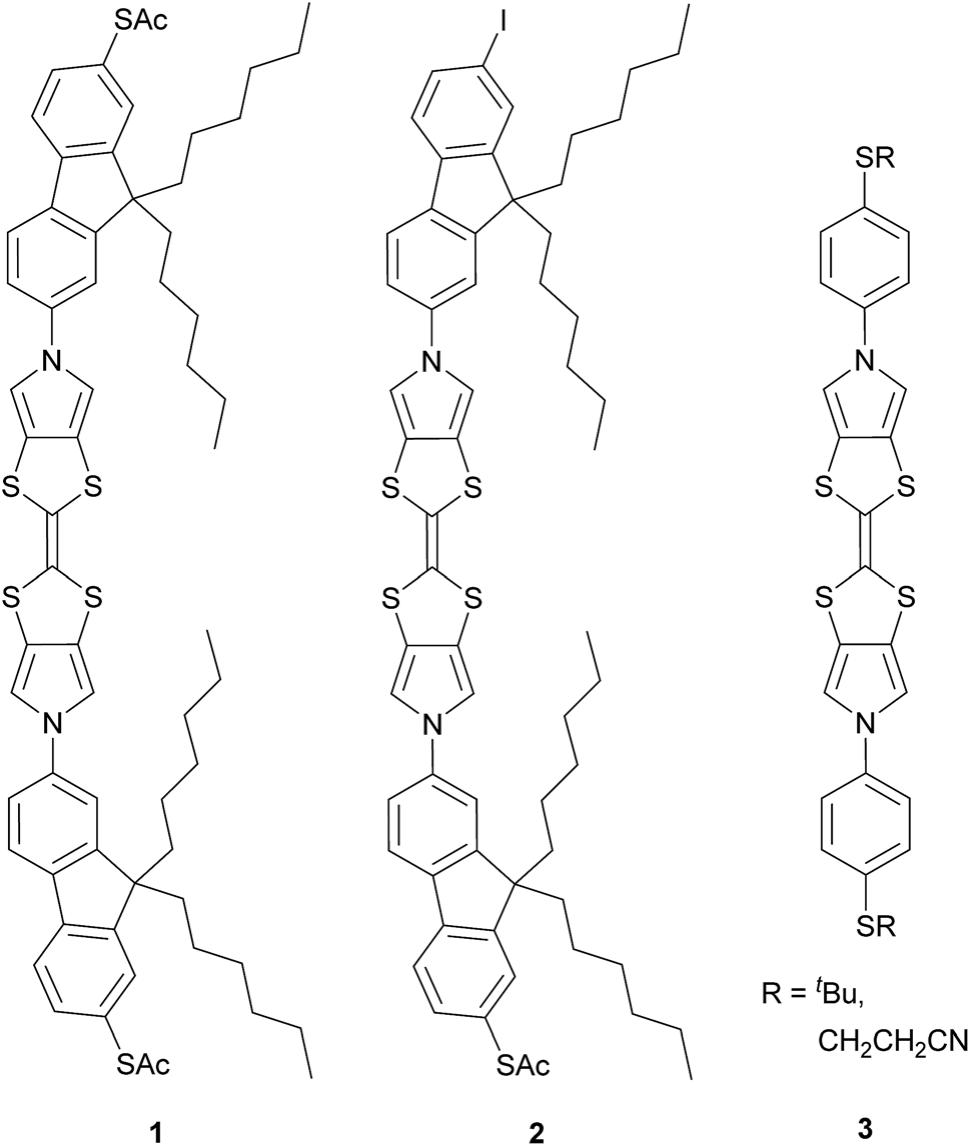
The BPTTF wires investigated in this work (**1** and **2**) and examples of precursors^36^ (**3**) of wires found to be insufficiently soluble for molecular conductance studies.

Here we report the synthesis of a conjugated molecular wire, **1**, in which a TTF unit forms part of the molecular backbone, *i.e.* the redox-active unit lies within the conductance pathway (Chart 1). Redox switching of this system changes the TTF unit between less-conjugated and more-conjugated states. The conductance and switching properties of the new wire were investigated using the STM-BJ method both with and without electrochemical control. The observed variation in molecular conductance is rationalised using DFT-based quantum transport simulations carried out using the GOLLUM code.^35^ The presence of an additional conductance feature is clarified by measurements of the analogous compound **2** (Chart 1) and computational investigations.

## Results and discussion

### Molecular design and synthesis

The synthesis of suitably functionalised TTF derivatives was based on the addition of aromatic substituents to a bis(pyrrolo)tetrathiafulvalene (BPTTF) unit *via* an Ullmann-type C–N coupling reaction.^36,37^ If a simple TTF is functionalised on each of the two five-membered rings the products are typically a mixture of *E* and *Z* isomers, which are difficult to separate and may interconvert during both purification and investigations.^38^ The use of a linear BPTTF unit means complications relating to this isomerism are avoided while retaining the desirable redox properties of the TTF core.

Our initial efforts to prepare suitable systems were based on BPTTFs functionalised with *para*-substituted benzene derivatives. The synthesis of some precursor systems (**3**, Chart 1) has been described previously.^36^ However, these systems were found to have very poor solubility, which precluded complete characterisation and molecular conductance studies. We therefore set about designing analogues with improved solubility. One consideration was to directly functionalise the benzene rings with alkyl or alkoxy chains. However, this would have resulted in systems in which a substituent lay *ortho* to the C–N bond, the anchoring moiety, or both. A substituent close to the C–N bond could induce a twist in the molecular backbone and consequently reduce conductance, while steric hindrance may also reduce the efficiency of the C–N coupling reaction. Another concern was that binding to gold substrates could be disrupted by the steric bulk of a neighbouring alkyl chain.

To alleviate these concerns we instead chose to work with fluorene as an alternative to substituted benzene rings. A popular bridging unit in donor–bridge–acceptor systems,^39,40^ fluorene is a planar aromatic system which can be easily functionalised at the 9-position with solubilising alkyl chains. This position was expected to be sufficiently distant from the BPTTF and anchoring moieties to avoid complications related to steric hindrance.

**Scheme 1:**
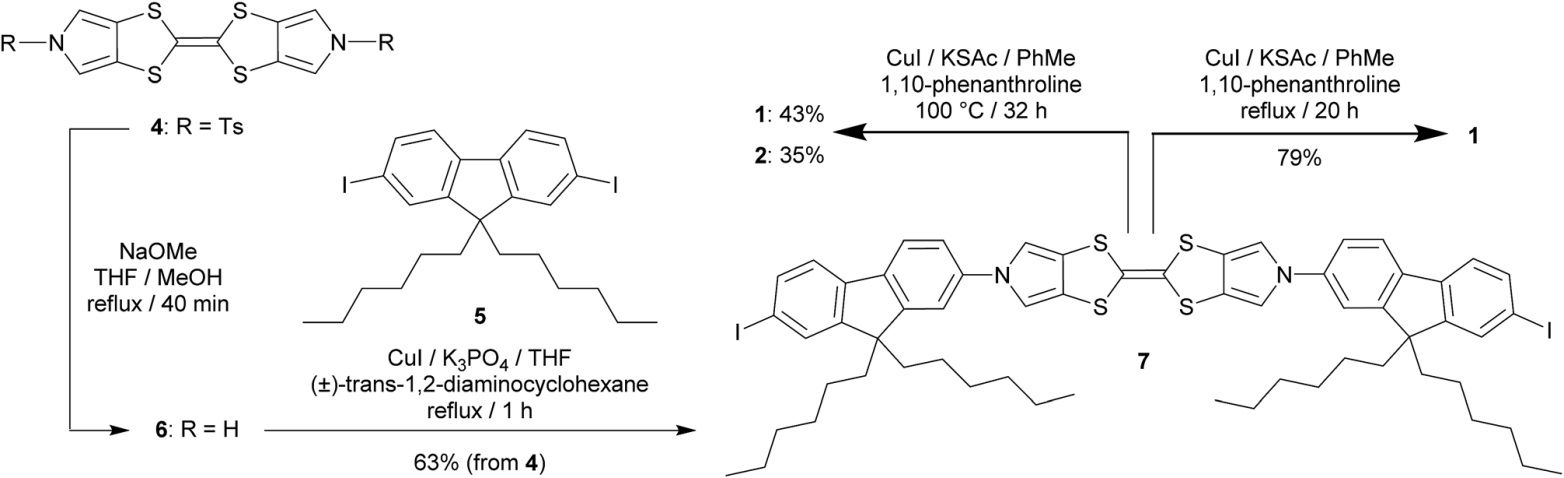
Synthesis of the BPTTF-based molecular wire **1** and analogue **2** bearing only a single thioacetate group.

The target molecule **1** (Chart 1) was prepared from the known, protected BPTTF **4** (ref. 36 and 41) and 2,7-diiodo-9,9′-dihexyl fluorene **5** (see ESI‡ for synthetic details) as shown in Scheme 1. The tosyl protecting groups of **4** were cleaved and the deprotected intermediate **6** was then reacted with an excess of **5** (to avoid the formation of oligomers) under Cu-catalyzed Ullmann-type coupling conditions^37^ to afford the diiodide **7** in 63% yield.‖
‖No evidence of appreciable oligomer formation was obtained. Compound **7** was observed to be somewhat unstable in solution, but was stable as a solid under ambient conditions. Attempts to introduce thioacetate anchoring groups by means of lithiation followed by treatment with sulfur then acetyl chloride were unsuccessful using both ^*n*^BuLi and ^*t*^BuLi. Instead, we utilised a Cu-catalyzed procedure^42^ which afforded **1** in 79% yield. By making small alterations to the reaction conditions (see ESI‡) it was also possible to isolate practical quantities of the monosubstituted analogue **2** (Chart 1).

### Electrochemical studies

**Fig. 1:**
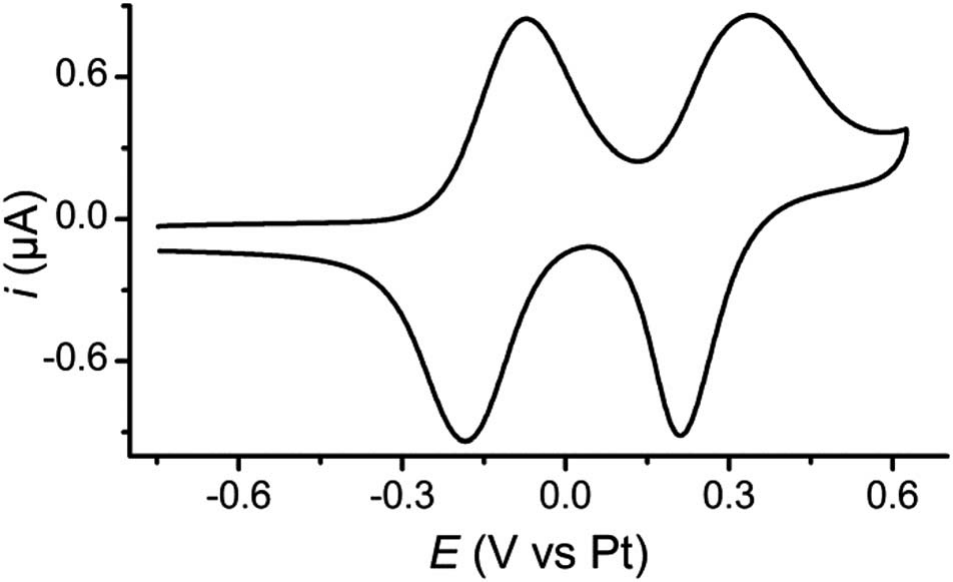
Cyclic voltammogram of **1** adsorbed on a Au(111) half-bead crystal working electrode with Pt reference and counter electrodes and 1-hexyl-3-methylimidazolium hexafluorophosphate (HMImPF_6_) ionic liquid supporting electrolyte. Scan rate: 50 mV s^–1^.

The redox behaviour of **1** was investigated using cyclic voltammetry (CV) which showed two reversible oxidations as expected for a TTF derivative, both for a monolayer and in solution. For a monolayer of **1** on a Au(111) half-bead crystal the redox potentials of these processes were *E*11/2 = –0.13 V and *E*21/2 = +0.28 V (both *vs.* Pt) (Fig. 1). Complementary studies on solutions of **1** and **2** in CH_2_Cl_2_ with a tetrabutylammonium hexafluorophosphate supporting electrolyte are detailed in the ESI (Fig. S9‡).

### Single molecule conductance studies

**Fig. 2:**
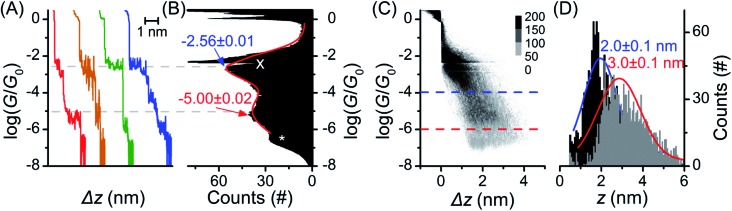
STM-BJ data from 1453 of 1488 (98%) junctions for **1**. (A) Four representative individual traces and (B) accumulated 1D histogram. The red curve in (B) is a 2-peak Gaussian fit; peak values are the fit results attributed to “full-length” (red) and “half-length” (blue) conformations (error is uncertainty in the least-squares fit). Dashed grey lines are guides for the eye showing peak locations in the 1D histogram. The white “X” marks an amplifier artifact excluded from Gaussian fit and the white “*” marks the peak from open-circuit noise. (C) 2D histogram of the same data. (D) plateau length histograms adjusted for snap-back^44^ with Gaussian fits showing most probable junction lengths (error is uncertainty in the fit) for trace lengths at the noise level (“full-length” conformation, grey histogram, red curve, dashed red line in (C)) and at a conductance of 10^–4.0^ *G*_0_ (“half-length” conformation, black histogram, blue curve, dashed blue line in (C)).

The single molecule conductance of molecular wire **1** was studied using the STM-BJ method^13^ (experimental conditions and data analysis are described in the ESI‡). Almost all traces (see examples in Fig. 2A) showed two distinct plateaus, which can be more easily observed when all traces are accumulated into a 1D conductance histogram (Fig. 2B). Overlaying all traces into a 2D histogram (Fig. 2C) shows that most junctions displayed two conductance features. The higher conductance feature (*ca.* 10^–2.6^ *G*_0_) corresponded to junction lengths of *ca.* 2.0 ± 0.1 nm, around half the length of **1**, as seen in the plateau length analysis (Fig. 2D and ESI‡). The lower conductance feature (*ca.* 10^–5.0^ *G*_0_) corresponded to longer junction lengths of *ca.* 3.0 ± 0.1 nm (Fig. 2D), *i.e.* closer to the length of **1** (3.05 nm in the ground state geometry from the DFT calculations below). As both conductance modes were present in nearly all traces (based on the 2D histogram), we concluded that they related to two distinct and sequential molecular conformations. We therefore hypothesise that the sulfur atoms present in the BPTTF unit can act as additional anchoring sites in the break junction measurements. Similar behaviour has been postulated for some exTTF systems^33^ and observed in an OPE wire containing a pyrimidine moiety.^43^ We propose that the higher conductance, shorter path-length feature is a Au–S–fluorene–BPTTF–Au junction (“half-length”), which, upon retraction of the STM tip, breaks to allow the desired Au–S–fluorene–BPTTF–fluorene–S–Au junction (“full-length”) to form (Fig. S12‡).

This hypothesis was tested by performing further STM-BJ experiments on wire **2** (see Fig. S10‡), a direct analogue of wire **1** in which one of the thioacetate anchoring groups is replaced by an iodide. With this modification we expected bridging events involving the full length of wire **2** would be much less likely, whereas events involving the central BPTTF unit would still be present. Indeed, the most prominent feature observed in Fig. S10B‡ is a peak at 10^–2.8^ *G*_0_ corresponding to an average junction length of 1.9 nm. The similarity of this feature and the high conductance feature observed for **1** indicates that they both relate to the same binding conformation. A weaker, less prominent feature with lower conductance and a longer junction length was also observed, possibly relating to weak anchoring from the iodide group.^45^ The proposed “half-length” Au–S–fluorene–BPTTF–Au junction was further supported by quantum transport simulations (*vide infra*).

**Fig. 3:**
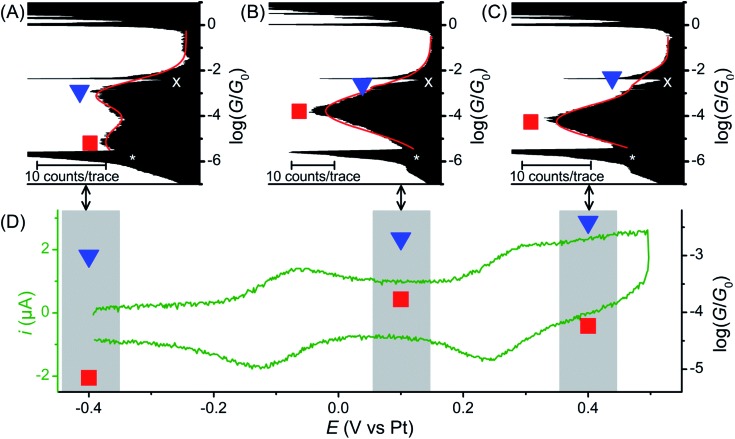
Electrochemically controlled STM-BJ data at 3 potentials: over 1600 traces each of **1** in the (A) neutral state (–0.4 V *vs.* Pt), (B) radical cation state (+0.1 V *vs.* Pt) and (C) dication state (+0.4 V *vs.* Pt). Red curves are 2-peak Gaussian fits for each distribution. The white “x” marks an amplifier artifact excluded from Gaussian fit and the white “*” marks the peak from open-circuit noise. (D) Representative *in situ* CV (green left axis and green curve) and conductance *vs.* redox state (black right axis). Red squares and blue triangles represent conductance features assigned to “full-length” and “half-length” conformations, respectively (errors are smaller than the size of the marks).

Further break junction measurements were conducted on wire **1** under electrochemical control^3^ (experimental details are described in the ESI‡). Three sample potentials (*E*) corresponding to the three redox states of the BPTTF unit were selected. Neutral state (*E* = –0.4 V *vs.* Pt) measurements showed reasonable agreement with those conducted without electrochemical control. In this case high- and low-conductance features were observed at 10^–3.0^ *G*_0_ and 10^–5.2^ *G*_0_, respectively (Fig. 3A, *cf.* 10^–2.6^ *G*_0_ and 10^–5.0^ *G*_0_ without electrochemical control). The small conductance variation may relate to differences in the experimental conditions, *e.g.* the change in the surrounding solvent environment.^46^

Upon oxidation to the radical cation state (*E* = +0.1 V *vs.* Pt) the conductance histogram shows a prominent feature with a molecular conductance of 10^–3.8^ *G*_0_ and a small shoulder at *ca.* 10^–3.0^ *G*_0_ (Fig. 3B). This indicates that the conductance along the full length of the molecule has increased by more than an order of magnitude, whereas that attributed to direct binding of one electrode to the BPTTF unit has not significantly changed. The dication state (*E* = +0.4 V *vs.* Pt) affords similar results with a somewhat less prominent shoulder and a slightly lower ‘full-length’ conductance of 10^–4.2^ *G*_0_ (still an order of magnitude larger than that of the neutral state, Fig. 3C). The reduced magnitude of the higher conductance features in the oxidised states suggests that the binding interaction between the STM tip and the BPTTF core is weaker in these cases, possibly due to the positive charge on the oxidised BPTTF unit. Our hypothesis that the high conductance feature relates to binding at the BPTTF is supported by the low variation of this conductance as the oxidation state of **1** is changed. If the high conductance pathway does not pass through the central bond of the BPTTF unit, it should change very little as the oxidation state of the BPTTF unit is varied and the extent of conjugation changes.

Focusing on the lower conductance feature, there is clear evidence to show that oxidation of the BPTTF unit can be used as a conductance switch, as illustrated in Fig. 3D. The increase in aromaticity upon oxidation from the neutral state to the radical cation should increase the delocalisation of electrons in the BPTTF unit and therefore enhance the conjugation along the wire, and indeed this results in a significant conductance increase (from 10^–5.2^ *G*_0_ to 10^–3.8^ *G*_0_). The magnitude of this conductance change is comparable to that previously reported for anthraquinone^6^ and naphthalenediimide^7^ based systems, and much larger than that achieved using a non-conjugated BPTTF switch.^8^ The subsequent reduction in molecular conductance upon oxidation to the dication (from 10^–3.8^ *G*_0_ to 10^–4.2^ *G*_0_) may relate to the increased rotational freedom about the central bond of the BPTTF unit (now a C–C single bond) which may somewhat disrupt the conjugation between the two halves of the BPTTF unit. The existence of three stable and reversibly accessible conductance states is a noteworthy feature, which could be exploited in logic gate applications. Whilst similar three-state behaviour is also seen in naphthalenediimide switches,^7^ the width of the potential window required to access the three different states is almost half the size in the case of **1**.

### Computational studies

**Fig. 4:**
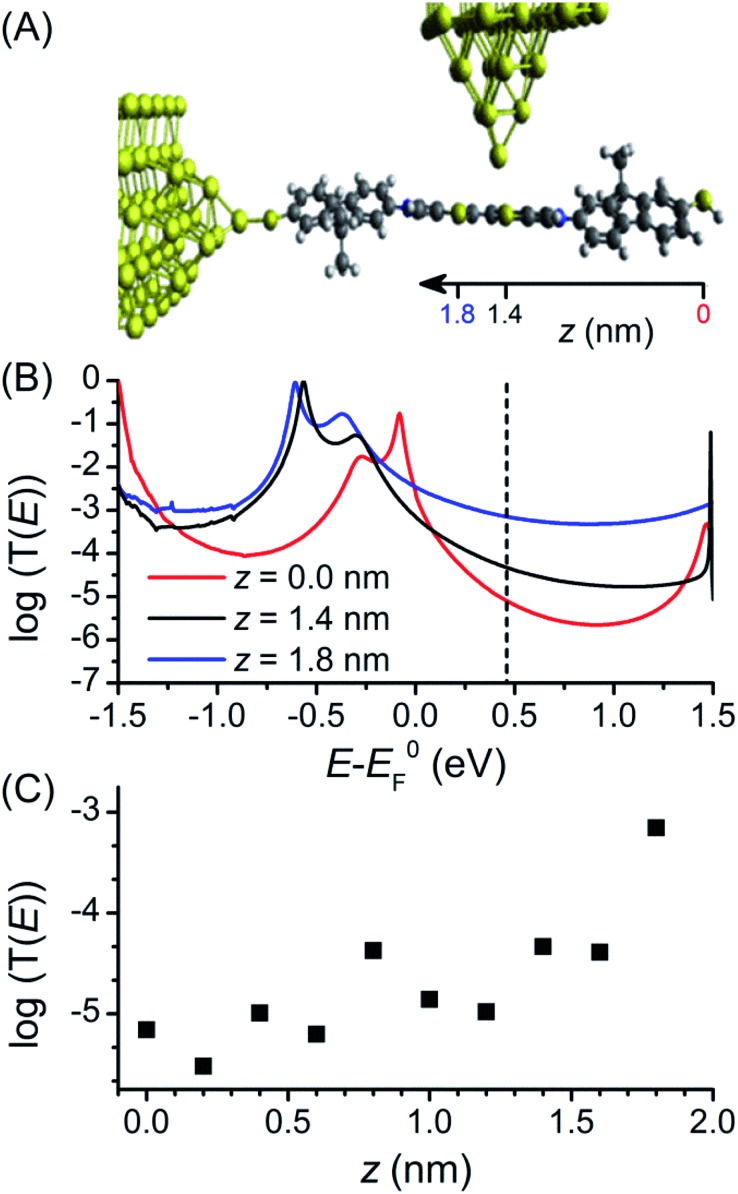
(A) Junction geometry for different electrode separations, the second electrode is moved a distance *z* along the axis of **1**. The approximate locations of selected *z* values are indicated. (B) Zero bias transmission coefficient for *z* = 0 nm, 1.4 nm and 1.8 nm (dashed line marks *E*_F_). (C) Conductance of **1** as a function of tip geometry *z* for *E*_F_ – *E*0F = 0.46 eV.

The first principles quantum transport code GOLLUM^35^ was used to calculate the conductance through **1** to assist interpretation of the experimental data (full details of the theoretical method can be found in the ESI‡). For these studies, methyl groups replace the hexyl chains attached to the fluorene units to reduce the computational expense, as they should play no role in molecular conductance.^18^ The acetyl protecting groups are also removed to correspond with the STM-BJ measurements. The ground state geometry of deprotected **1** was first connected to a gold electrode *via* one of the terminal thiol groups with an optimal binding distance of 2.4 Å (the binding energy was calculated to be 0.9 eV). To explore the high and low conductance features, the second electrode was shifted along the molecular backbone by a distance *z* as shown in Fig. 4A. As the second thiol group is not bound to gold for all geometries its hydrogen atom was retained throughout. Binding energy calculations showed that binding to the central BPTTF unit was optimum at 2.55 Å above the sulfur atoms at distances *z* = 1.4 and 1.8 nm from the second terminal thiol with a value of 0.35 eV.

A Hamiltonian describing the ‘extended molecule’ was produced using the density functional code SIESTA^47^ and the zero bias transmission coefficient *T*(*E*) was calculated for a range of values of *z* (keeping the tip distance above the molecule constant). Plots of three values, *z* = 0, 1.4 and 1.8 nm, are shown in Fig. 4B (in all cases spin polarised *T*(*E*) calculations were performed); the first of these represents a fully extended junction and the latter two values correspond to junction conformations in which the tip binds to a BPTTF sulfur atom on either side of the central double bond. For the fully extended junction (*z* = 0 nm, red) the HOMO resonance sits close to the DFT predicted Fermi energy (*E*0F) which is typical for thiol anchored molecules. The value of the conductance evaluated at *E*0F is 10^–2.7^ *G*_0_, which is significantly higher than the value measured for the neutral state. This can be attributed to the known problems of DFT transport calculations in accurately determining the position of the true Fermi energy, *E*_F_.^48^ While corrections can be made,^49^ these are difficult to perform for thiol-anchored molecules where the molecule changes upon binding to gold. Therefore, we treat *E*_F_ as a parameter and use the experimental data to determine its position. At *E*_F_ – *E*0F = 0.46 eV the value of conductance matches the experimental value of 10^–5.2^ *G*_0_.

The conductance was then calculated for different values of *z*. For *z* = 1.8 nm (Fig. 4B, blue curve), where the conductance pathway does not pass through the central double bond, the conductance at *E*_F_ – *E*0F = 0.46 eV is 10^–3.1^ *G*_0_ (*cf.* the experimentally observed high conductance feature under electrochemical conditions at *G* = 10^–3.0^ *G*_0_). The transmission curve for this “half-length” pathway through the molecule shows the HOMO resonance is shifted to lower energy and the off-resonance value is higher across the HOMO–LUMO gap. For *z* = 1.4 nm (Fig. 4B, black curve), where the conductance pathway does pass through the central double bond, the predicted conductance at *E*_F_ – *E*0F = 0.46 eV is considerably lower, only 10^–4.3^ *G*_0_. A corresponding conductance feature is not observable in the experimental data; it may be that the nature of the binding between the tip and the BPTTF unit is more complex than the modelled conformations. The transmission curves in Fig. 4B show a similar trend for a wide range of values of *E*_F_ – *E*0F, not just the experimentally fitted value of 0.46 eV.

Fig. 4C shows the value of conductance at *E*_F_ – *E*0F = 0.46 eV for these and additional values of *z*. When the tip is contacted to the fluorene part (*z* < 0.8 nm) the conductance is low, but when it contacts to the central BPTTF unit (*z* > 1 nm) the conductance increases and at a value of *z* = 1.8 nm agrees with the high conductance measurement. This value of *z* corresponds to a conductance pathway that does not pass through the central bond of the BPTTF unit and therefore should not be significantly affected by conjugation changes. This supports the attribution of the weak, high conductance shoulder peaks observed experimentally for the oxidised states of **1** to binding geometries of this type.

Electrochemical control of the low conductance conformation (*z* = 0 nm) was modelled using a previously developed charge double layer model^7^ (full details of this simulation can be found in the ESI‡). Here *E*_F_ is not treated as a free parameter and the neutral state is fixed by agreement with the experimental value of 10^–5.2^ *G*_0_. The position of the double layer is then modified so that the number of electrons (*N*) on the molecule (relative to the neutral state) decreases until the radical cation state is reached (*N* = –1). Table 1 shows that the conductance increases significantly, and further removal of electrons to afford the dication state (*N* = –2) causes the conductance to decrease slightly, which qualitatively follows the trend of the experimental data. In addition to the conjugation changes discussed above, the initial increase in conductance can be attributed to pinning of the spin split HOMO resonances at *E*0F when the molecule becomes charged. As the molecule becomes more positively charged the spin splitting increases (Fig. S17‡), lowering the conductance. The overestimation of the conductance in this model can be explained by the appearance of ‘gateway’ orbitals typical of thiol anchor groups.^50^

**Table 1:** Conductance as a function of the redox state of wire **1**

Redox state	*N* ^*a*^	Calculated conductance, log(*G*/*G*_0_)	Experimental conductance,^*b*^ log(*G*/*G*_0_)
Neutral	0	–5.10	–5.17 ± 0.03
Radical cation	–1	–0.46	–3.781 ± 0.006
Dication	–2	–0.60	–4.239 ± 0.004

^*a*^Number of electrons on **1** relative to the neutral state.

^*b*^Values given are those assigned to the “full-length” junction conformation; (error is uncertainty in the least-squares fit).

## Conclusions

A conjugated single molecular switch based on BPTTF, **1**, has been designed, synthesised and characterised. The conductance properties of **1** were studied using the STM-BJ method and DFT-based quantum transport simulations. The molecular conductance of **1** is observed to increase by over an order of magnitude upon oxidation from the neutral state to the radical cation, then decrease slightly upon further oxidation to the dication. A qualitatively similar trend is obtained from simulations in which the charge on **1** is adjusted using a charge double layer model. The experimental results are complicated somewhat by the existence of alternative binding conformations that are attributed to the sulfur atoms within the BPTTF unit, as evidenced by analysis of junction lengths, studies of the analogous system **2** with only one thiol anchor and computational studies of binding conformations. Efforts to improve the system and prevent this unwanted binding are currently underway in order to more easily exploit the redox properties of BPTTF in single molecule switches.
