# Supplementary material for: Electrochemical control of the single molecule conductance of a conjugated bis(pyrrolo)tetrathiafulvalene based molecular switch
Source: Chem Sci. 2017 Jun 23;8(9):6123–30. doi: 10.1039/c7sc02037f (PMC5625590; doi:10.1039/c7sc02037f)
Supplement: Supplementary file 1 [file SC-009-C7SC02037F-s001.pdf]

Electronic Supplementary Material (ESI) for Chemical Science.
This journal is © The Royal Society of Chemistry 2017

Electronic Supplementary Information

Electrochemical control of the single molecule conductance of a conjugated bis(pyrrolo)tetrathiafulvalene based molecular switch

Luke J. O'Driscoll,[a] Joseph M. Hamill,[*b] Iain Grace,[c] Bodil W. Nielsen,[a] Eman Almutib,[c] Yongchun Fu,[b] Wenjing Hong,[bd] Colin J. Lambert[*c] and Jan O. Jeppesen[*a]

[a] Department of Physics, Chemistry and Pharmacy, University of Southern Denmark, Campusvej 55, DK-5230, Odense M, Denmark.

[b] Department of Chemistry and Biochemistry, University of Bern, Freiestrasse 3, CH-3012, Bern, Switzerland.

[c] Department of Physics, Lancaster University, Lancaster, United Kingdom, LA1 4YB.

[d] Present Address: State Key Laboratory of Physical Chemistry of Solid States, College of Chemistry and Chemical Engineering, Collabora-tive Innovation Center of Chemistry for Energy Materials, Xiamen University, 361005 Xiamen, China

Email: Jan O. Jeppesen: joj@sdu.dk

Colin J. Lambert: c.lambert@lancaster.ac.uk

Joseph M. Hamill: joseph.hamill@dcb.unibe.ch

* Corresponding authors

Contents:

Synthesis:

General Procedures S3

Experimental Procedures and Characterisation S3

Selected NMR Spectra S11

Selected MALDI-MS Spectra S15

Measurements:

CV Procedures and Data S19

STM-BJ Procedures and Data Analysis S20

EC-STM-BJ Procedures and Data Analysis S24

Computational Studies:

Junction Geometries S28

Varying Molecular Charge S31

References: S34

Synthesis:

General Procedures

Anhydrous MeOH and THF were distilled from Mg and molecular sieves (4 Å), respectively, immediately prior to use. Other anhydrous solvents were allowed to stand over molecular sieves (4 Å) for at least three days prior to use. All reagents were standard grade and used as received, except the protected BPTTF **4**[S1, S2] which was prepared according to a previously published procedure. Thin-layer chromatography (TLC) was carried out using aluminium sheets pre-coated with silica gel 60F (Merck F_{254}); visualisation was achieved using UV light (254 nm) or I_2. Column chromatography used silica gel (ROCC 40-60 µm); eluent mixes are given as v/v ratios. Melting points (mp) were determined on a Büchi 535 melting point apparatus and are uncorrected. ^{1}H and ^{13}C NMR spectra were recorded at room temperature on a Bruker AVANCE III spectrometer (400 MHz and 100 MHz, respectively). Chemical shifts are quoted on the δ scale and coupling constants (J) are expressed in Hertz (Hz). Samples were prepared using solvents purchased from Sigma Aldrich and all spectra were referenced using the residual solvent peak as an internal standard. Matrix Assisted Laser Desorption Ionisation (MALDI) mass spectra were recorded on a Bruker Autoflex III Smartbeam instrument. Elemental analyses were performed by Atlantic Microlabs, Inc., Atlanta, Georgia.

Experimental Procedures and Characterisation

Compound **5** was prepared based on literature procedures previously used to prepare similar alkylated diiodofluorenes,[S3, S4] as shown in Scheme S1.

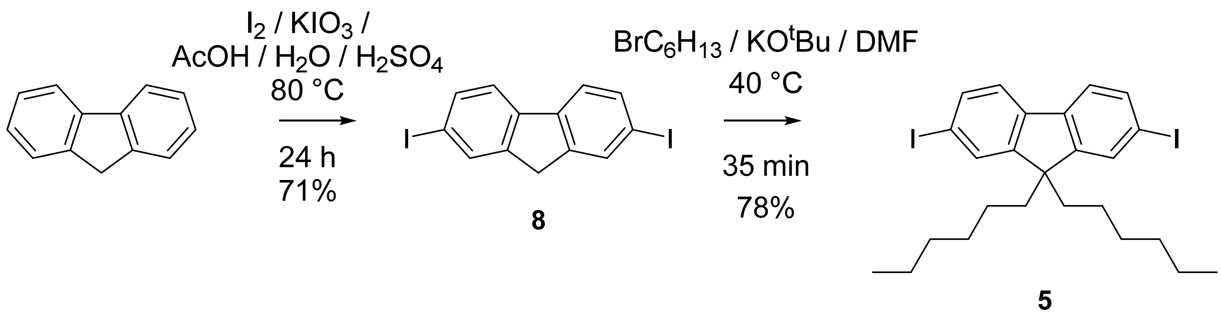

Scheme S1. Synthesis of 2,7-diiodo-9,9'-dihexyl fluorene (**5**) from fluorene.

2,7-Diiodofluorene (8)[S3]:

Fluorene (9.97 g, 60.0 mmol) was added to a 50:4:1 mixture of AcOH:H_2O:H_2SO_4 (660 mL) and heated to 80 °C. After the fluorene had dissolved, KIO_3 (5.14 g, 24.0 mmol, 0.4 eq.) and I_2 (16.6 g, 65.4 mmol, 1.09 eq.) were added and the reaction was stirred at 80 °C overnight. After cooling to room temperature, the off-white precipitate was filtered off and washed with saturated aqueous Na_2CO_3 (500 mL) and deionised (DI) H_2O (500 mL), then dried *in vacuo*. Recrystallisation from CH_2Cl_2 and subsequent drying *in vacuo* afforded **8** as off-white needles (17.8 g, 71%), mp 217.5 – 218.5 °C (lit.[S3] 216 – 217 °C), ^{1}H NMR (400 MHz, $CDCl_3$): δ 7.87 (d, *J* = 0.6 Hz, 2H), 7.70 (dd, *J* = 8.0 Hz, 0.8 Hz, 2H), 7.49 (d, *J* = 8.0 Hz, 2H), 3.83 (s, 2H); ^{13}C NMR (100 MHz, $CDCl_3$): δ 145.0, 140.6, 136.2, 134.3, 121.7, 92.6, 36.5; MS-MALDI *m*/*z*: 417.897 ($M^{+\bullet}$).

2,7-Diiodo-9,9'-dihexyl fluorene (5):

This synthesis was based on a previously reported procedure for the dioctyl analogue.[S4] 2,7-Diiodofluorene **8** (15.0 g, 35.9 mmol, 1 eq.) was dispersed in DMF (250 mL) in an oven-dried flask under N_2. 1-Bromohexane (15.1 mL, 108 mmol, 3 eq.) and KO^{t}Bu (10.1 g, 90.0 mmol, 2.5 eq.) were added to the stirred mixture which was then heated to 40 °C for 1 h. The reaction was quenched by addition of DI H_2O (200 mL) and allowed to cool to room temperature, after which the mixture was extracted with ether (300 mL, then 200 mL). The combined organic layers were washed with DI H_2O (3 × 200 mL) and brine (200 mL), dried ($MgSO_4$) and the solvent removed *in vacuo*. The resulting yellow oil was purified by column

chromatography (10 cm Ø, 1000 mL SiO_2; eluent: cyclohexane), yielding **5** as a yellow solid (16.4 g, 78%), mp 59.0 – 60.0 °C (lit.[S5] 58.5 – 60 °C), ^{1}H NMR (400 MHz, $CDCl_3$) δ 7.67 – 7.62 (m, 4H), 7.40 (dd, J = 7.7, 0.8 Hz, 2H), 1.94 – 1.84 (m, 4H), 1.19 – 1.08 (m, 4H), 1.08 – 0.98 (m, 8H), 0.78 (t, J = 7.1 Hz, 6H), 0.64 – 0.50 (m, 4H); ^{13}C NMR (100 MHz, $CDCl_3$) δ 152.7, 139.9, 136.2, 132.2, 121.6, 93.3, 55.7, 40.3, 31.6, 29.7, 23.8, 22.7, 14.2; MS-MALDI *m*/*z*: 586.124 ($M^{+\bullet}$), Anal. Calcd for $C_{25}H_{32}I_2$: C, 51.21; H, 5.50. Found: C, 51.44; H, 5.45.

The following synthetic steps are described in the main text, but are summarised again here in Schemes S2-S5.

Bis(pyrrolo[3,4-*d*])tetrathiafulvalene (6):

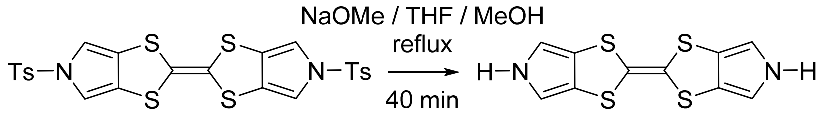

4 **6**

Scheme S2. Deprotection of bis(*N*-tosylpyrrolo-[3,4-*d*])tetrathiafulvalene.

6 was prepared according to our previously published route.[S1, S2] Full purification was not performed prior to the subsequent Ullmann coupling reaction on account of previously observed instability of deprotected pyrrolotetrathiafulvalenes.[S1]

Bis(*N*-tosylpyrrolo-[3,4-*d*])tetrathiafulvalene[S1, S2] **4** (675 mg, 1.14 mmol) was dispersed in anhydrous THF (45 mL) and anhydrous MeOH (45 mL) in an oven-dried flask under N_2, then degassed (N_2, 15 min). NaOMe (25% solution in MeOH, 7.9 mL, 35 mmol, 30 eq.) was added and the reaction heated under reflux for 40 min. The reaction was cooled to room temperature and concentrated to ca. 30 mL *in vacuo* before DI H_2O (150 mL) was added, causing a yellow precipitate to form. This was isolated by filtration then dried under vacuum to provide **6** as a yellow solid, which was used directly in the next step without further purification.

Diiodide BPTTF wire precursor (7):

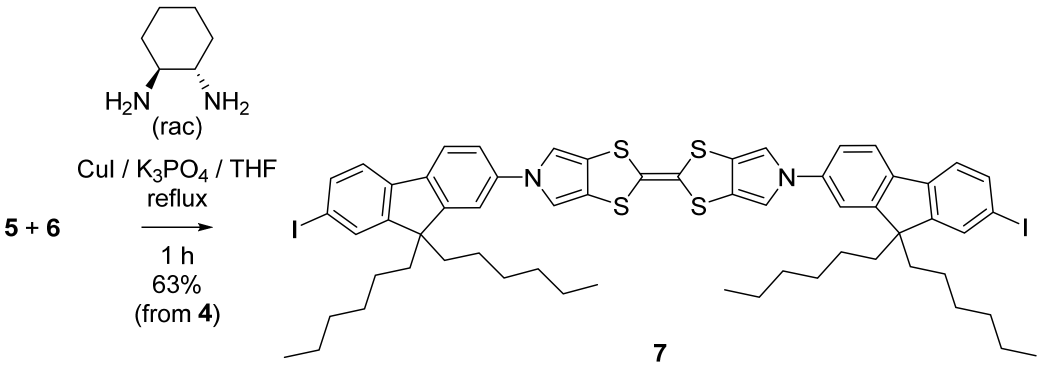

Scheme S3. Functionalisation of BPTTF with hexyl-functionalised fluorene moieties.

The crude BPTTF **6** from the previous step was dried for ca. 1 h under vacuum, then compound **5** (6.70 g, 11.4 mmol, 10 eq. based on **4**), CuI (435 mg, 2.29 mmol, 2 eq. based on **4**) and K_3PO_4 (849 mg, 4.00 mmol, 3.5 eq. based on **4**) were added to the same flask, which was dried under vacuum for ca. 1 h, then placed directly under N_2. The mixture was dissolved/dispersed in anhydrous THF (70 mL) then degassed (N_2, 15 min) before (±)-*trans*-1,2-diaminocyclohexane (0.54 mL, 4.46 mmol, 3.9 eq. based on **4**) was added and the mixture heated to vigorous reflux. The yellow-green reaction mixture turned pea-green upon heating. After 1 h TLC[1] indicated that **6** had been consumed and the reaction was allowed to cool to room temperature overnight. The reaction mixture was filtered through a pad of Celite 545 to remove insoluble impurities; the filter was washed with CH_2Cl_2 to ensure the yellow product-containing band was collected. The filtrate was washed with aqueous NaOH (3 × 75 mL[2], 1 wt. %) and DI H_2O (3 × 75 mL), before the organic layer was dried ($MgSO_4$) then concentrated *in vacuo* to give a yellow solid. This crude product was redissolved in CH_2Cl_2 and concentrated onto Celite (ca. 50 mL), then purified by column chromatography (10 cm Ø, 900 mL SiO_2; eluents: cyclohexane until elution of **5** was complete[3], then 4:1 cyclohexane/CH_2Cl_2 until the slightly impure product had eluted) by placing the Celite

[1] The product **7** has a very similar R_f to starting material **5** in 1:1 cyclohexane/CH_2Cl_2, but a very low R_f in cyclohexane only.
[2] The aqueous layer was purple in the first extraction.
[3] The recovered **5** is of sufficient purity to be re-used.

mixture directly on top of the column. A second chromatography column (5 cm Ø, 300 mL SiO_2; eluent: 8:1 cyclohexane/CH_2Cl_2) was used to complete the purification (again loading the crude material onto Celite) and **7** was isolated as a yellow solid (866 mg, 63% over 2 steps based on **4**) with traces of grease[4], mp 243.0 – 244.0 °C (decomp.), ^{1}H NMR (400 MHz, CD_2Cl_2[5]): δ 7.75 – 7.66 (m, 6H), 7.46 (d, J = 7.9 Hz, 2H), 7.38 – 7.28 (m, 4H), 7.01 (s, 4H), 2.02 – 1.92 (m, 8H), 1.15 – 0.98 (m, 24H), 0.77 (t, J = 7.1 Hz, 12H), 0.66 – 0.56 (m, 8H); ^{13}C NMR (100 MHz, CD_2Cl_2): δ 153.8, 152.9, 140.6, 136.7, 132.9, 122.6, 122.0, 121.5, 120.7, 119.7, 115.3, 111.7, 93.0, 56.3, 40.8, 32.1, 30.2, 24.4, 23.1, 14.3[6]; MS-MALDI m/z: 1198.658 ($M^{+•}$), 1072.630 ($(M$–I+H$)^{+•}$), 946.640 ((M–2I+2H)$^{+•}$), 600.197 ((1/2M+H)$^{+}$[7]); Anal. Calcd for $C_{60}H_{68}I_2N_2S_4$ + 3 × –CH_2–: C, 60.96; H, 6.01; N, 2.26; S, 10.33. Found: C, 61.05; H, 6.00; N, 2.23; S, 10.63.

BPTTF wire (1):

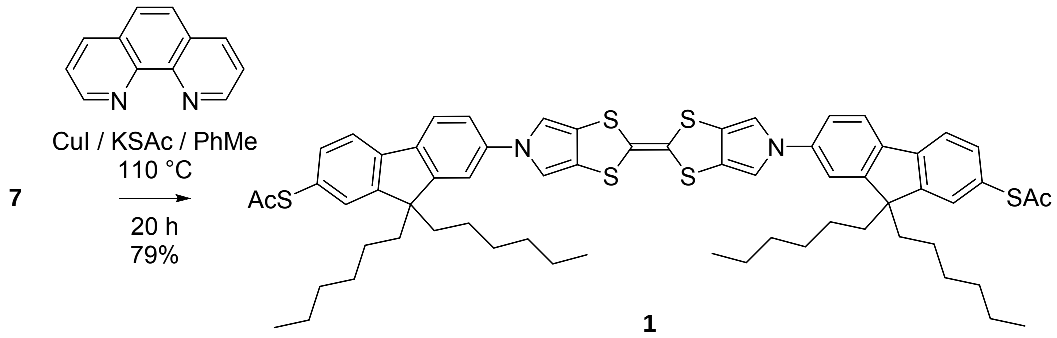

Scheme S4. Synthesis of the targeted BPTTF-based wire **1** by conversion of iodine substituents to thioacetate groups.

This reaction was conducted in a flask covered with aluminium foil to exclude light. The diiodide precursor **7** (125 mg, 0.104 mmol, 1 eq.) was dissolved in anhydrous toluene (5 mL) in an oven-dried flask under N_2 and degassed (N_2, 1 h). CuI (8 mg, 0.04 mmol, 0.4 eq.),

[4] Further purification was not conducted due to the poor solution stability of the compound.
[5] The compound was found to be more stable in CD_2Cl_2 than in $CDCl_3$ and this solvent also prevented overlap of the solvent signal with aromatic signals.
[6] Two aromatic signals are missing or overlapping. The poor solution stability of the compound meant that it was difficult to obtain a good quality ^{13}C NMR spectrum even with an extended scan time, pulse delay and efforts to exclude light.
[7] For a more detailed assignment of the MALDI data, see the MS section below.

1,10-phenanthroline (15 mg, 0.083 mmol, 0.8 eq.) and KSAc (36 mg, 0.31 mmol, 3 eq.) were added and the mixture was heated under reflux for 20 h. The reaction was cooled to room temperature and ether (7.5 mL) and DI H_2O (7.5 mL) were added. The organic phase was separated and the aqueous phase was washed with ether (2 × 10 mL). All of the organic phases were combined and dried (Na_2SO_4) before the solvent was removed *in vacuo*. The resulting yellow-brown oil was purified by column chromatography (4 cm Ø, 200 mL SiO_2; eluents: cyclohexane was used to prepare the column, then 150 mL 2:1 cyclohexane/CH_2Cl_2 was used to begin elution of the product before switching the eluent to 1:1 cyclohexane/CH_2Cl_2) to afford **1** as a yellow solid (90 mg, 79%). As small quantities of grease and cyclohexane were visible in the ^{1}H NMR spectrum, the compound was recrystallised from ^{n}BuOH yielding yellow crystals (50 mg, 44%) of analytically pure **1**, mp 194.0 – 195.5 °C (decomp.), ^{1}H NMR (400 MHz, CD_2Cl_2[8]) δ 7.77 (d, *J* = 8.1 Hz, 2H), 7.76 – 7.71 (m, 2H), 7.44 – 7.38 (m, 4H), 7.38 – 7.31 (m, 4H), 7.03 (s, 4H), 2.42 (s, 6H), 2.10 – 1.90 (m, 8H), 1.17 – 0.98 (m, 24H), 0.76 (t, *J* = 7.0 Hz, 12H), 0.72 – 0.58 (m, 8H); ^{13}C NMR (100 MHz, CD_2Cl_2) δ 194.8, 153.7, 152.3, 142.1, 140.6, 138.9, 134.0, 129.8, 127.3, 122.6, 121.8, 120.7, 119.7, 115.4, 111.7, 56.3, 40.8, 32.1, 30.6, 30.2, 24.4, 23.1, 14.3[9]; MS-MALDI m/z: 1094.878 ($M^{+\bullet}$), 1052.647 $(M\text{–}Ac+H)^{+\bullet}$, 548.277 $(1/2M+H)^{+}$[10]; Anal. Calcd for $C_{64}H_{74}N_2O_2S_6$: C, 70.16; H, 6.81; N, 2.56; S, 17.56. Found: C, 70.10; H, 6.82; N, 2.62; S, 17.64.

[8] Using this solvent rather than $CDCl_3$ prevented overlap of the residual solvent signal with aromatic signals.
[9] One peak is overlapping or not observed.
[10] For a more detailed assignment of the MALDI data, see the MS section below.

Monothiolated BPTTF wire (2):

A slightly modified scale up of the above synthesis of **1** did not result in full conversion even after an extended reaction time. We took advantage of this observation to isolate the monosubstituted analogue **2**.

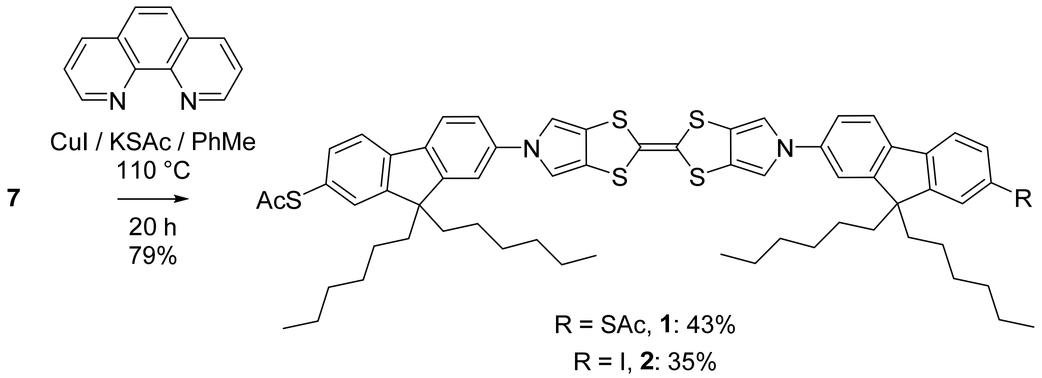

Scheme S5. Scale-up of the above synthesis of wire **1** did not reach completion and also afforded monosubstituted analogue **2**.

This reaction was conducted in a flask covered with aluminium foil to exclude light. A flask containing the diiodide precursor **7** (760 mg, 0.63 mmol, 1 eq.) was dried under vacuum overnight then placed directly under N_2. Anhydrous toluene (30 mL) was added and the resulting solution was degassed (N_2, 20 min). CuI (26 mg, 0.14 mmol, 0.22 eq.), 1,10-phenanthroline (46 mg, 0.26 mmol, 0.41 eq.) and KSAc (217 mg, 1.90 mmol, 3 eq.) were added and the mixture was heated to 100 °C for 32 h. The reaction was cooled to room temperature and ether (100 mL) and DI H_2O (100 mL) were added. The organic phase was separated and the aqueous phase was washed with ether (2 × 40 mL). All of the organic phases were combined and dried (Na_2SO_4) before the solvent was removed *in vacuo*. The resulting yellow-brown oil was purified by column chromatography (6 cm Ø, 400 mL SiO_2; eluents: cyclohexane was used to prepare the column, then 2:1 cyclohexane/CH_2Cl_2 was used until a weak by-product band between unreacted **7** and **2** eluted before switching the eluent to 1:1 cyclohexane/CH_2Cl_2 to elute **2**, then **1**). Characterisation of **1** (295 mg, 43%) was in agreement with that reported above. The isolated **2** was passed through a second chromatography column (3 cm Ø, 200 mL SiO_2; eluent: CH_2Cl_2) to remove traces of grease,

affording **2** as a yellow solid (251 mg, 35%), mp 186.0 – 187.5 °C (decomp.), ^{1}H NMR (400 MHz, CD_2Cl_2[11]) δ 7.80 – 7.65 (m, 5H), 7.46 (d, *J* = 8.0 Hz, 1H), 7.42 – 7.28 (m, 6H), 7.02 (s, 2H), 7.01 (s, 2H), 2.42 (s, 3H), 2.07 – 1.90 (m, 8H), 1.17 – 0.98 (m, 24H), 0.77 (2 × t, *J* = 7.0 and 7.2 Hz, 12H), 0.70 – 0.55 (m, 8H); ^{13}C NMR (100 MHz, CD_2Cl_2) δ 194.7, 153.8, 153.7, 152.8, 152.3, 142.1, 140.6, 138.9, 136.7, 134.0, 132.8, 129.8, 127.3, 122.63, 122.61, 122.0, 121.8, 121.5, 120.7, 119.7, 115.4, 115.2, 111.7, 93.0, 56.31, 56.28, 40.81, 40.76, 32.1, 30.6, 30.19, 30.16, 24.40, 24.36, 23.14, 23.13, 14.33, 14.32[12]; MS-MALDI m/z: 1146.853 ($M^{+\bullet}$), 1104.681 $(M–Ac+H)^{+\bullet}$, 1020.669 $(M–I+H)^{+\bullet}$, 600.197 (1/2*M*+H, I-substituted)$^{+}$, 548.307 (1/2*M*+H, SAc-substituted)$^{+}$[13]; Anal. Calcd for $C_{62}H_{71}IN_2OS_5$: C, 64.90; H, 6.24; N, 2.44; S, 13.97. Found: C, 65.14; H, 6.34; N, 2.30; S, 13.71.

[11] Using this solvent rather than $CDCl_3$ prevented overlap of the residual solvent signal with aromatic signals.
[12] Several peaks are overlapping or not observed, presumably due to the near-symmetrical nature of the molecule.
[13] See discussion of MALDI data for compounds **1** and **7** - comparable additional peaks are also observed in this case, but not included in the discussion for brevity.

Selected NMR Spectra

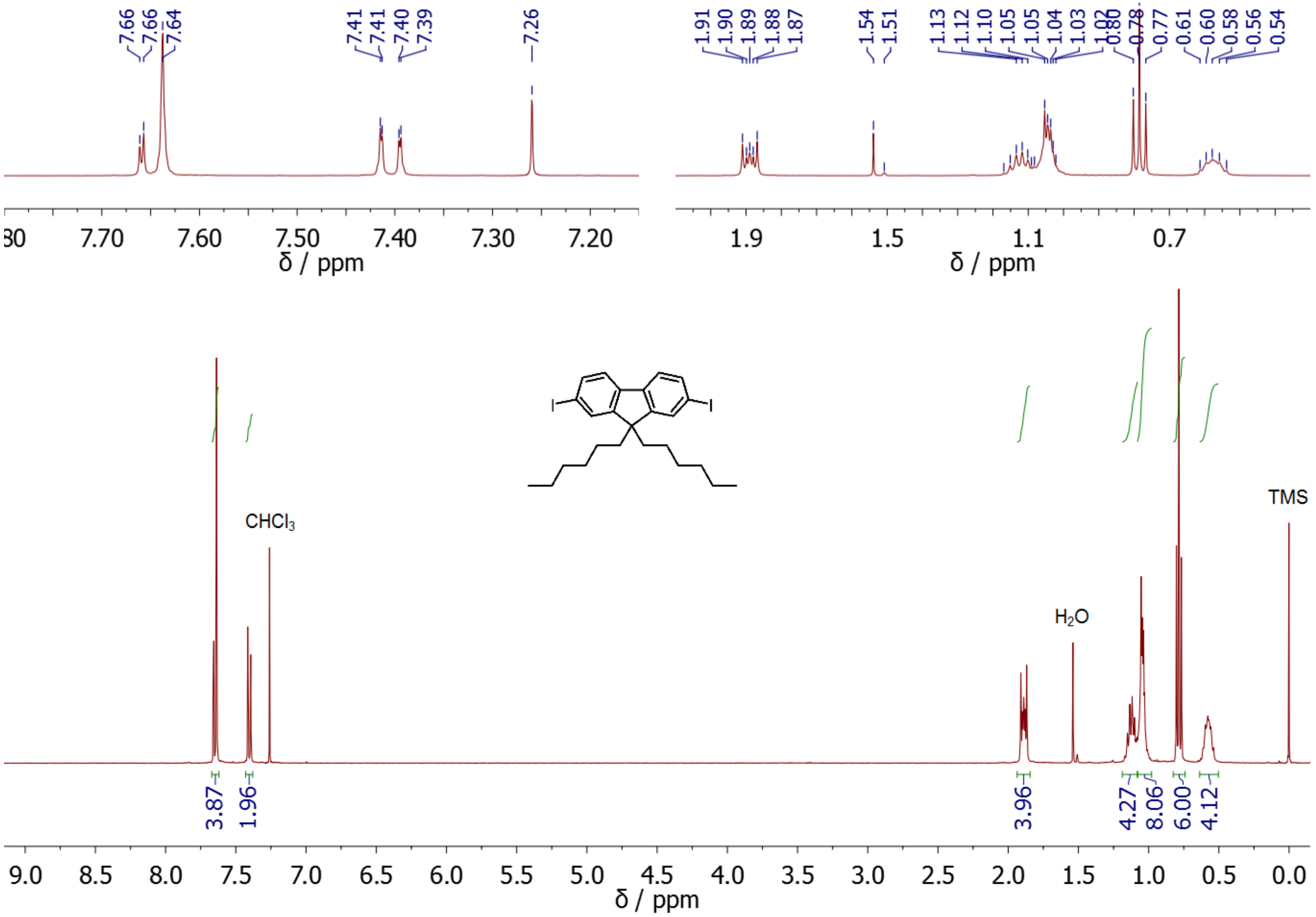

Fig. S1. Below: ^1H NMR spectrum of **5** (400 MHz, $CDCl_3$, RT). Above: Partial spectra highlighting peak locations.

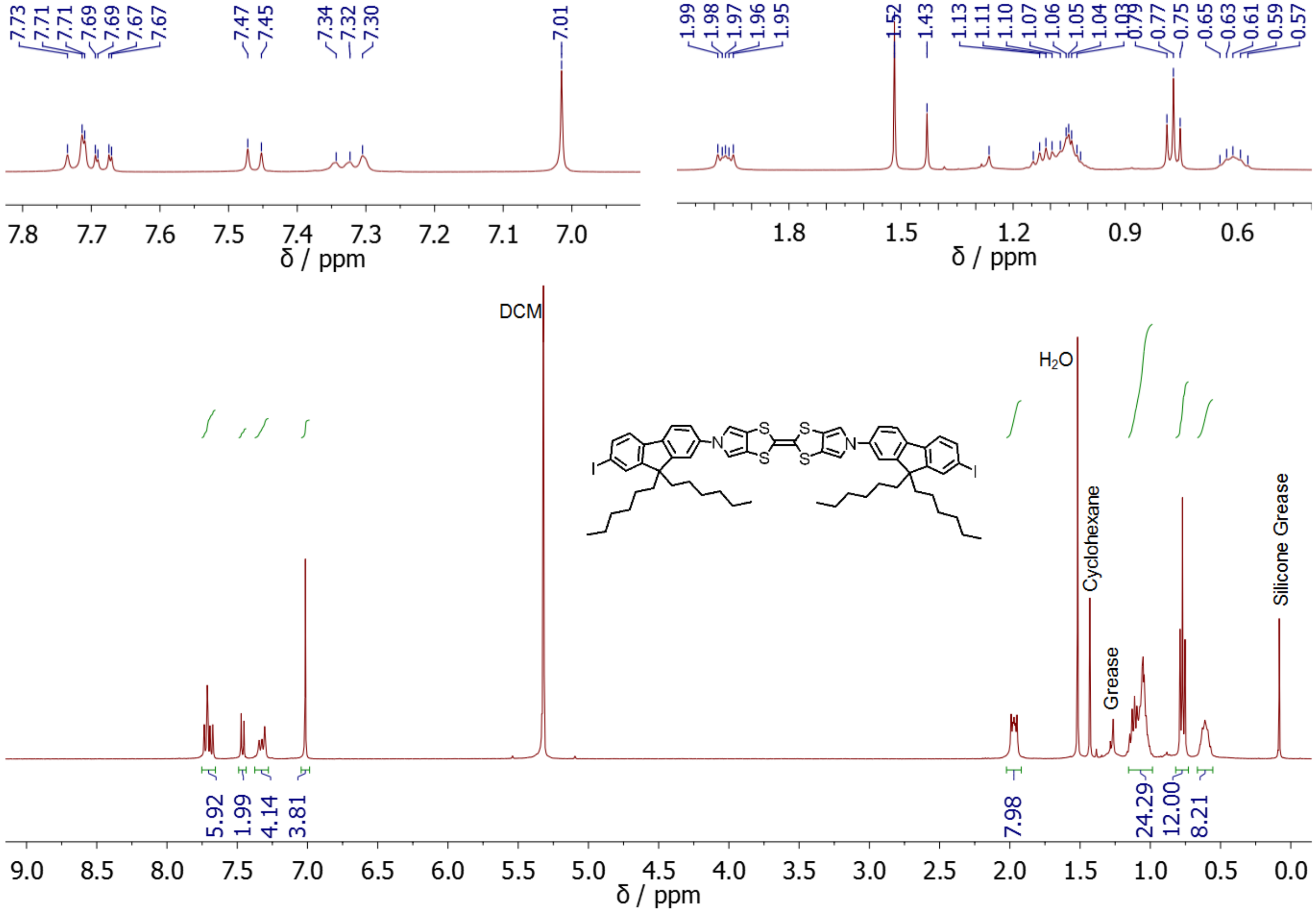

Fig. S2. Below: ^{1}H NMR spectrum of **7** (400 MHz, CD_2Cl_2, RT). Above: Partial spectra highlighting peak locations.

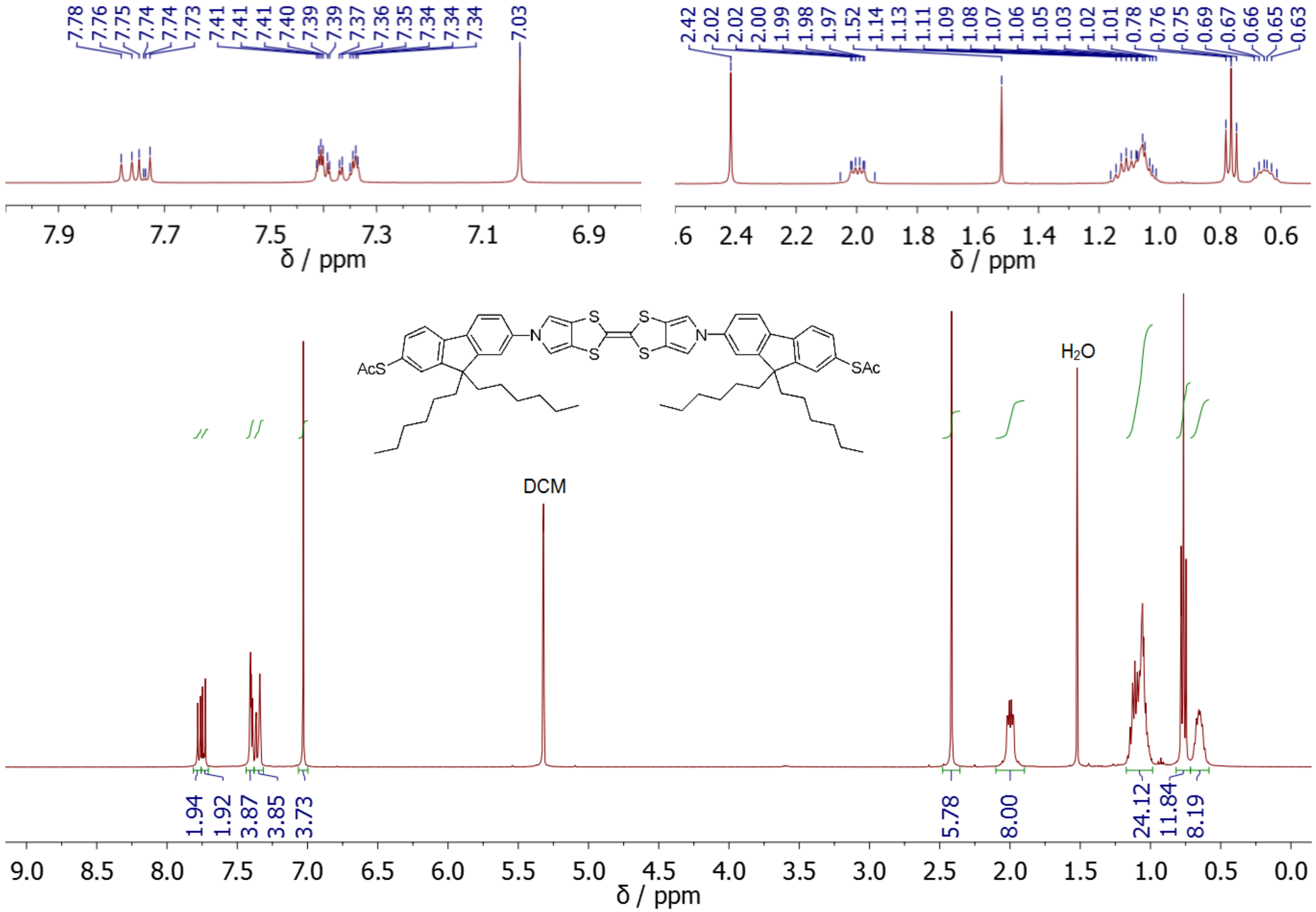

Fig. S3. Below: ^{1}H NMR spectrum of **1** (400 MHz, CD_2Cl_2, RT). Above: Partial spectra highlighting peak locations.

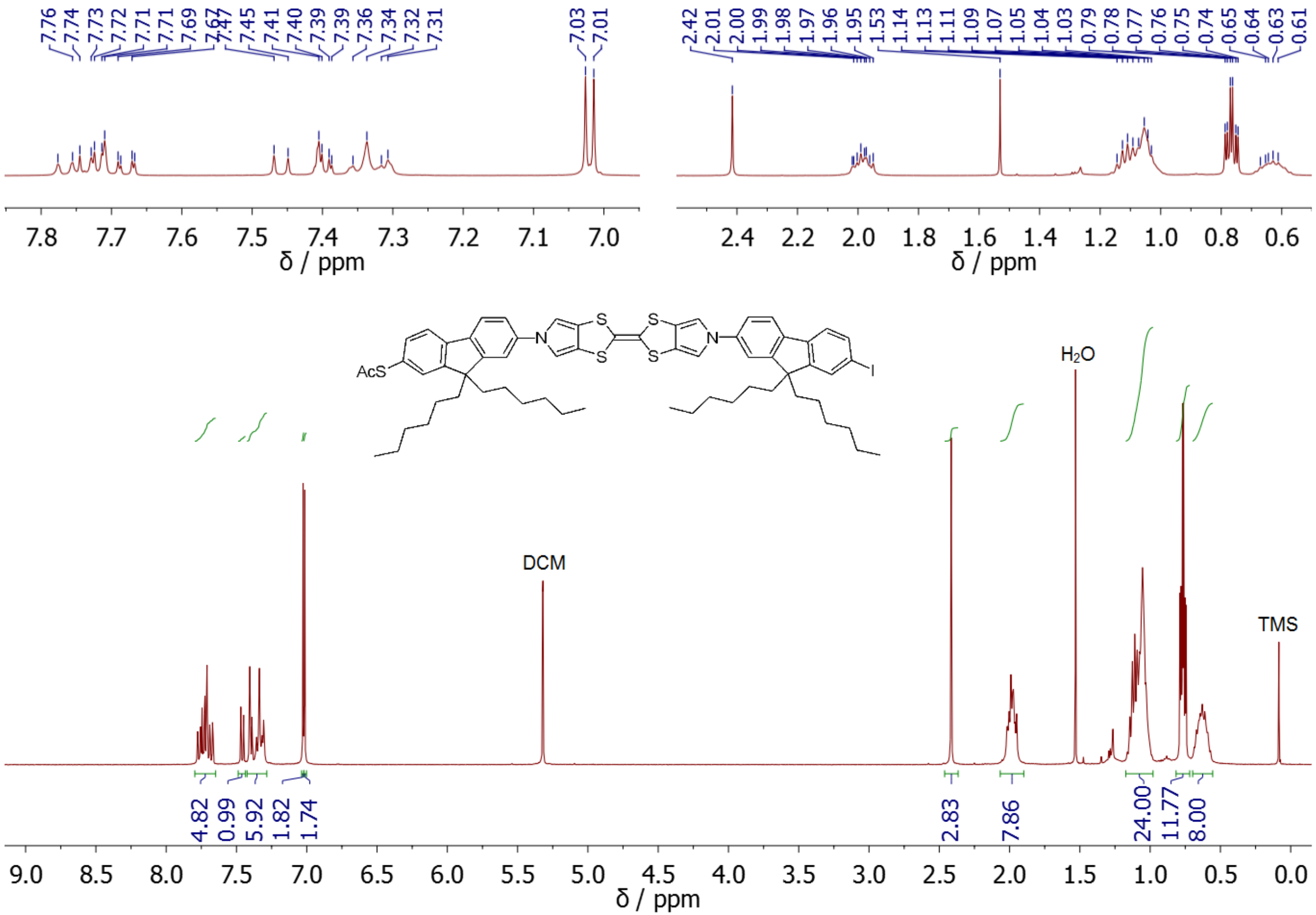

Fig. S4. Below: ^{1}H NMR spectrum of **2** (400 MHz, CD_2Cl_2, RT). Above: Partial spectra highlighting peak locations.

Selected MALDI-MS Spectra

Compounds **1**, **2** and **7** afforded many peaks in their MALDI mass spectra. These appeared to relate to several fragmentations and rearrangements including substitution of I (**2**, **7**) or Ac (**1**, **2**) with H, loss of CO (**1**, **2**), cleavage of the central bond of the BPTTF unit affording a (½M + H)$^+$ fragment* (Fig. S5a) and rearrangement of one or both dihexylfluorene moieties to phenanthrene (Fig. S5b). Peaks featuring a fluorene to phenanthrene rearrangement are consistently accompanied by another peak at m/z + 34, which presumably relates to an unidentified intermediate of the rearrangement process. Assignments for compounds **1** and **7** are summarised in Figs S6 and S7 (**7**) and S8 (**1**). Compound **2** afforded similar spectra, which are excluded here for brevity.

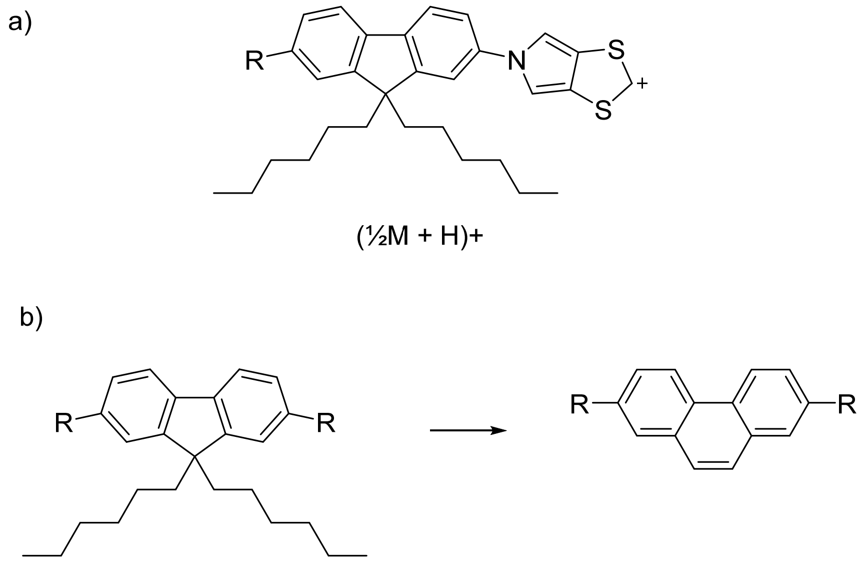

Fig. S5. Proposed fragmentations and rearrangements observed in the MALDI-MS of **1**, **2** and **7**: a) Expected structure of the fragment assigned to (½M + H)$^+$; b) Rearrangement of dihexylfluorene to phenanthrene.

* Distinguished from the possible M^{2+} peak which would appear in the same region by integer intervals between isotope peaks.

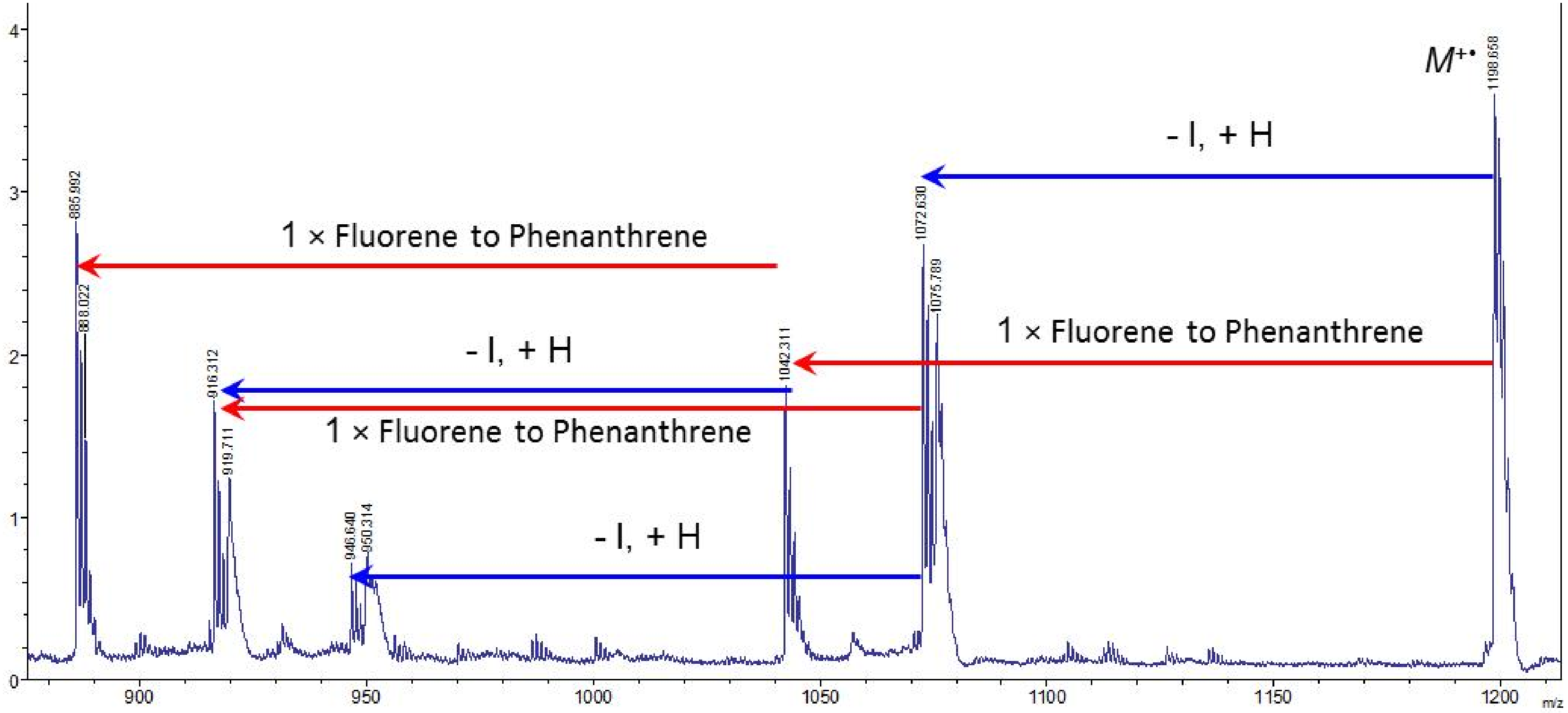

Fig. S6. Partial MALDI-MS for **7**, with some proposed fragmentations and rearrangements highlighted.

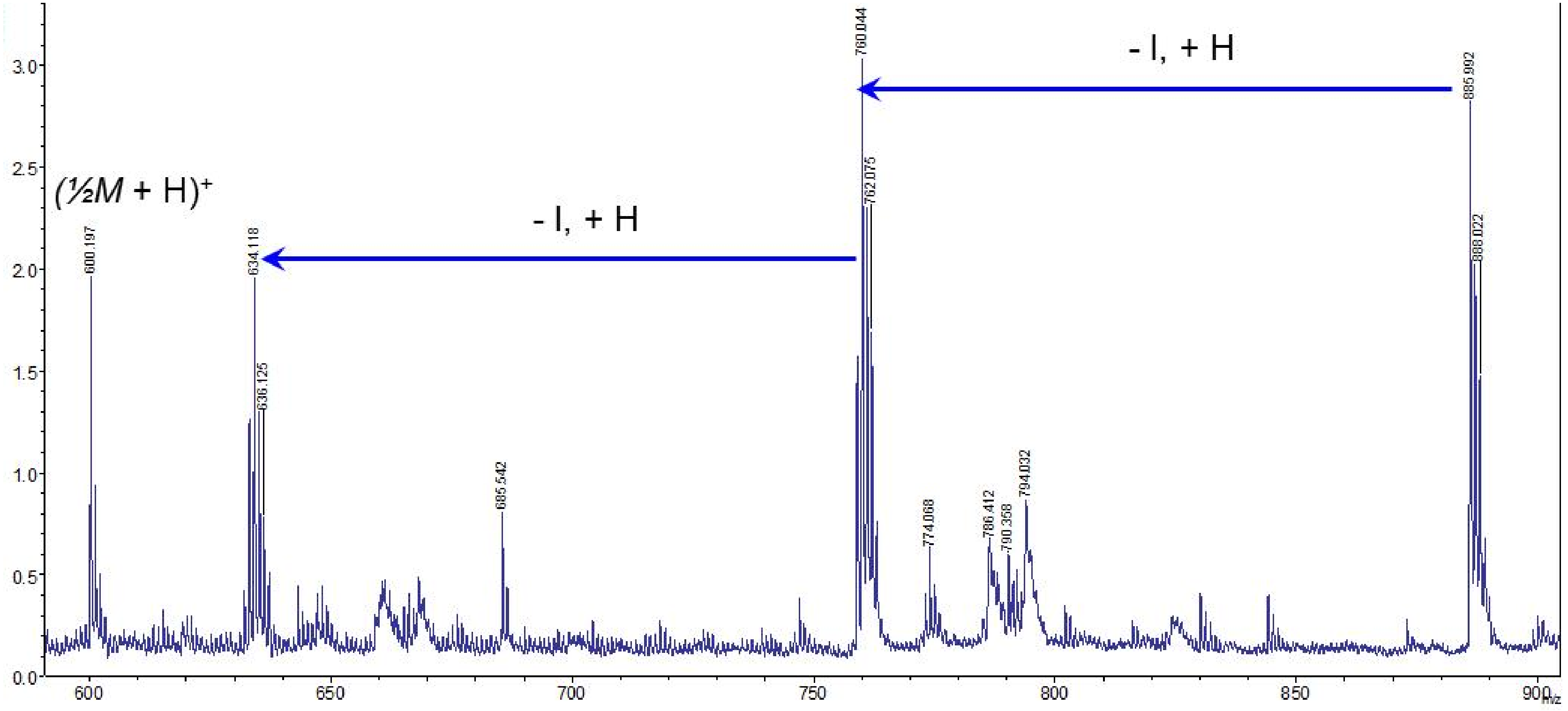

Fig. S7. Additional partial MALDI-MS for **7**, with some proposed fragmentations and rearrangements highlighted.

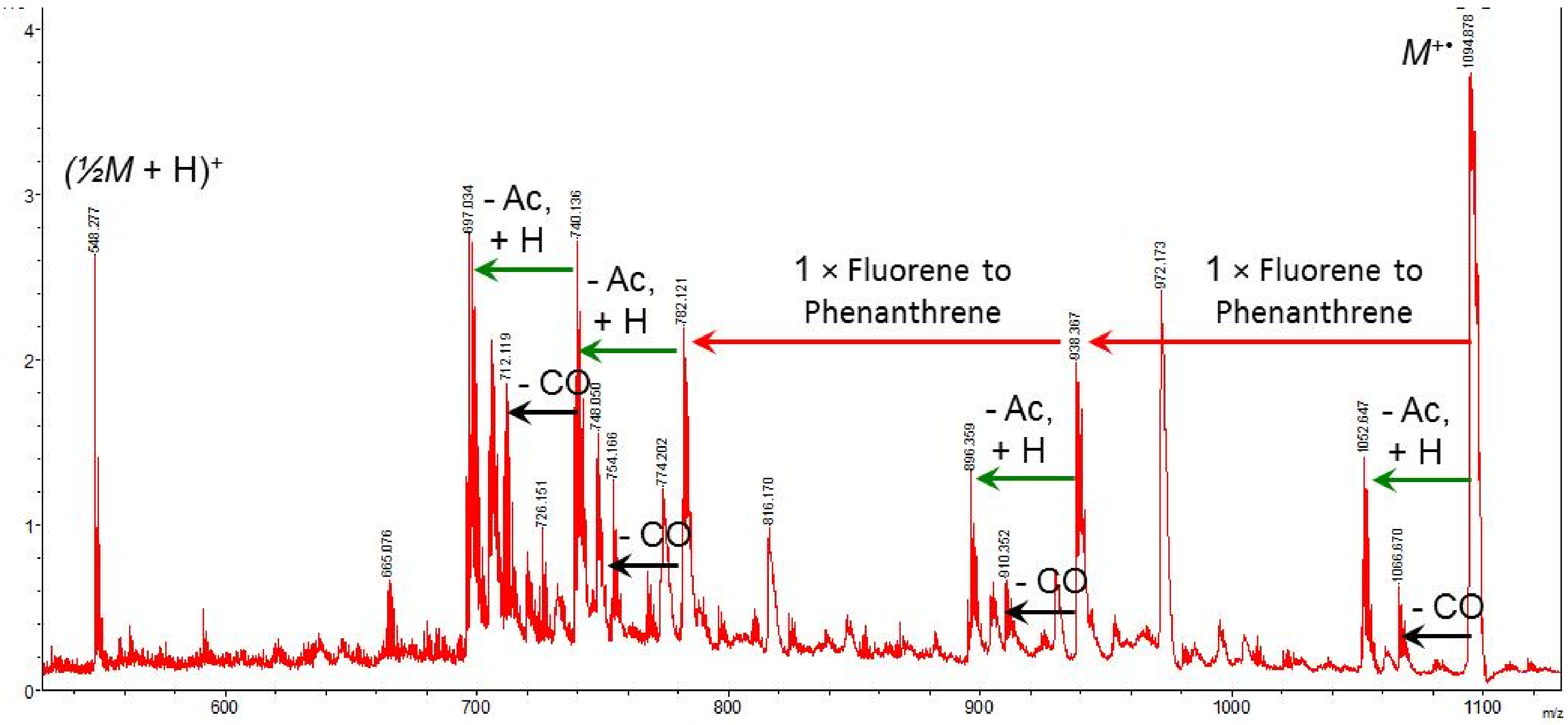

Fig. S8. Partial MALDI-MS for **1**, with some proposed fragmentations and rearrangements highlighted.

Measurements:

CV Procedures and Data

Solution Measurements

Cyclic voltammetry was conducted using an Autolab PGSTAT10 potentiostat and a Bioanalytical Systems Inc. C3 cell stand. Measurements used a 1 mM analyte solution in CH_2Cl_2 with 0.1 M tetrabutylammonium hexafluorophosphate ($^{n}Bu_4NPF_6$) as the supporting electrolyte, which was degassed (N_2) for 10-15 min prior to measurements. The setup utilised a glassy carbon working electrode, platinum wire counter electrode and a Ag/$AgNO_3$ (in MeCN with 0.1 M $^{n}Bu_4NPF_6$) reference electrode with a scan rate of 100 mV s^{-1} at room temperature. The reported data is given relative to ferrocene, for which a voltammogram was obtained under the same conditions prior to recording that of the compound of interest. Under these conditions, two reversible oxidations were observed for both **1** and **2**, as expected for TTF derivatives (Fig. S9). The redox potentials of these processes were $\boldsymbol{E}^{\mathbf{1}}_{½}$ = 0.00 V and $\boldsymbol{E}^{\mathbf{2}}_{½}$ = +0.47 V (vs Fc/Fc^{+}) for **1** and $\boldsymbol{E}^{\mathbf{1}}_{½}$ = 0.00 V and $\boldsymbol{E}^{\mathbf{2}}_{½}$ = +0.46 V (vs Fc/Fc^{+}) for **2**. The small structural difference between these two compounds has negligible effect on their redox behavior.

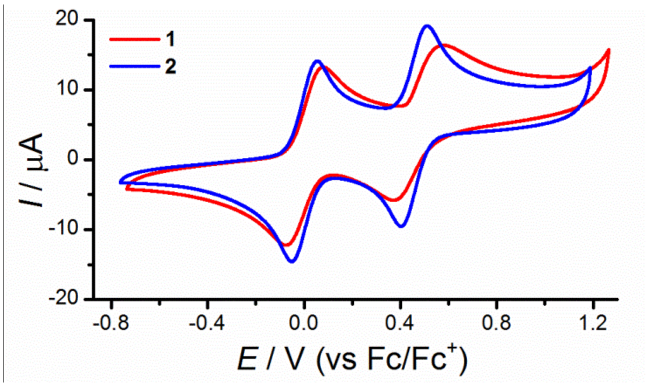

Fig. S9. Cyclic voltammogram of the BPTTF wires **1** and **2**. Conditions: 1 mM analyte in CH_2Cl_2 with 0.1 M $^{n}Bu_4NPF_6$ (supporting electrolyte). Electrodes: working: glassy carbon; counter: Pt; reference: Ag/$AgNO_3$ (in MeCN with 0.1 M $^{n}Bu_4NPF_6$). Scan rate: 100 mV s^{-1}.

Monolayer Measurements

Electrochemistry was performed in a standard electrochemical cell to characterise the monolayer of **1**. A flame-annealed Au(111) half-bead crystal was soaked overnight in a saturated solution of **1** in THF. A cyclic voltammogram (CV) was measured in a 1-hexyl-3-methylimidazolium hexafluorophosphate ($HMImPF_6$) ionic liquid electrolyte utilising Pt reference and counter electrodes at room temperature in an Ar-purged electrochemical cell. Fig. 1 in the main text shows two reversible redox events, the first at $E^1_{½}$ = –0.13 V and the second at $E^2_{½}$ = +0.28 V (both vs. Pt).

STM-BJ Procedures and Data Analysis

Experimental procedure

Break junctions were measured using the scanning tunneling microscopy break junction (STM-BJ) technique.[S6] A solution of 0.1 mM **1** in 1:4 THF:mesitylene (v/v) was prepared. A flame-annealed Au half-bead crystal was used for the STM substrate and a 0.25 mm etched Au tip was prepared. A Kel-F liquid cell was used to immerse the prepared substrate in 150 µL of the solution of **1**. Break junctions were created at a rate of 87 nm s^{-1}; example traces are shown in Fig. 2A of the main text.

Data analysis

1D histogram

98% of 1488 (1453) traces were accumulated into a 1D histogram (Fig. 2B, main text). 2% of traces were removed because they did not fit the basic criteria of a break junction: they did not start above 10^0 G_0 (G_0 = $2e^2h^{-1}$ = 77 µS) and did not finish below 10^{-6} G_0 within 6 nm. The 1D histogram showed peaks whenever a break junction trace had a plateau at a particular conductance value. For instance, there was a narrow peak at 10^0 G_0 immediately before the final Au-Au bond broke and there was a broad peak when the junction was

completely open, representing the lower limit of our experimental sensitivity (below $10^{-6.0}$ G_0). There were two more peaks in the 1D histogram which resulted from the presence of **1** in the break junction. A two-peak Gaussian model was fit to the 1D histogram in the full range between $10^{-0.5}$ G_0 and $10^{-5.5}$ G_0 and the centres of the two peaks were determined to be $10^{-2.556\pm0.008}$ G_0 and $10^{-4.996\pm0.018}$ G_0.

2D histogram

All 1453 traces were overlaid to create a 2D conductance-displacement histogram (2D histogram, Fig. 2C of the main text). The 2D histogram confirmed that nearly all junctions had two conductance plateaus: one at ~$10^{-2.6}$ G_0 and another at ~$10^{-5.0}$ G_0.

Plateau length histogram

The length of each trace from immediately after the breaking of the Au-Au junction at $10^{-0.3}$ G_0 until the trace approached the noise level at $10^{-5.5}$ G_0 represented the length of the molecular junction. These lengths were accumulated into a plateau length (PL) histogram (grey histogram, Fig. 2D of the main text) which offered an explanation for the two peaks in the 1D histogram. The PL histogram had a Gaussian distribution with a mean of 2.52 nm (with an error of ±0.05 nm in the fit). After adjusting for the snap back of the Au-Au junction by adding 0.5 (±0.1) nm,[S7] the mean value of the PL histogram was a good estimate for the full length of the molecule, from terminal S atom to terminal S atom, of 3.05 nm[*]. Very few junctions were shorter than 1.5 nm. The PL histogram thus suggested that most junctions achieved the full length of the molecule and therefore the two peaks in the 1D histogram result from single molecules achieving at least two meta-stable conformations, each with separate conductance values, in a single junction cycle[†]. The 2D histogram also confirmed that the conductance plateaus at ~$10^{-5.0}$ G_0 corresponded to the molecular conductance when the Au-**1**-Au junction was fully extended and the molecule was oriented perpendicular

[*] DFT-calculated ground state geometry.

[†] Although longer and shorter junctions were also measured, these account for only a handful of events, as shown in the PLH. These are presumably other, less likely junction conformations which occur much less frequently than the predominant ~3 nm junctions with 2 plateau features.

to the gold substrate within the junction. Furthermore, the conductance plateaus at $10^{-2.5}$ G_0 corresponded to displacements of about half the molecular length (2.0 ± 0.1 nm after adjusting for snap back). These "half-length" PL histograms were calculated by determining the length of each trace when it reached a conductance of $10^{-4.0}$ G_0, instead of the full conductance range of $10^{-5.5}$ G_0. This new PL histogram (black histogram, Fig. 2D of the main text) can be attributed to a meta-stable "half-length" bonding configuration in which one or more of the sulfur atoms in the BPTTF interact with one of the Au leads. The BPTTF sulfur atoms lie 1.4 or 1.8 nm from each terminal sulfur (see Computational Studies), so a junction involving these sites would be expected to be slightly shorter than, but comparable to, the observed value of 2.0 ± 0.1 nm.

STM-BJ with monothiol (2)

To test the hypothesis that the central BPTTF unit interacts with one of the Au electrodes during break junction experiments on **1**, the analogous wire **2**, with an iodide group replacing one of the thioacetate anchor groups, was synthesised (see above). As this molecule has only a single (protected) thiol anchor, it was expected that "full-length" molecular junctions would not form, because the iodide was not anticipated to interact significantly with the Au electrode. On the contrary, if the suspected interaction between a Au electrode and BPTTF can take place, the previously observed high conductance feature should still be observable. Fig. S10 shows the results of an STM-BJ experiment following the same procedures as above. A prominent plateau and peak are visible in the 2D and 1D histograms, respectively (Figs S10A and S10B), but a weak plateau/peak is also present. The 2-peak fit of the 1D histogram showed the peaks to be at $10^{-2.80\pm0.018}$ and $10^{-4.27\pm0.06}$ G_0 and the plateau length histogram fit associated these conductance values with electrode displacements of 1.86±0.12 and 3±3 nm, respectively. The prominent short distance, high conductance feature is similar to the feature observed in the STM-BJ results of wire **1** and we interpreted the cause to be identical – an interaction between one of the Au electrodes and the BPTTF unit

affording a “half-length” conformation. Surprisingly, the iodide end group appeared to interact weakly in the junction to afford the second feature, however, this long distance, low conductance feature was far less probable than junctions only involving the shorter, higher conductance feature. We note that there are limited examples of the use of iodide as an anchoring group in break junction studies.[S8, S9]

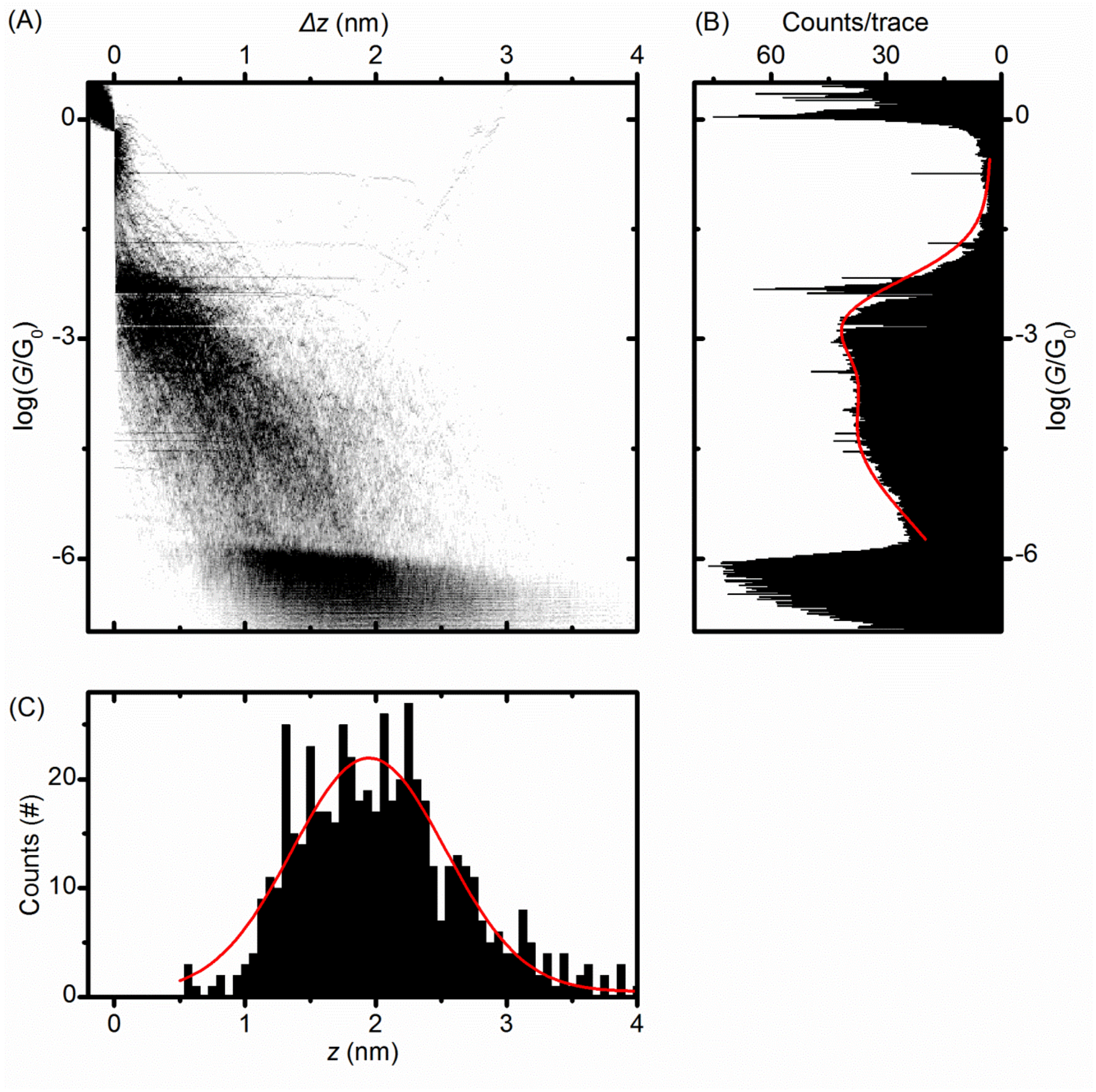

Fig. S10. STM-BJ results for 0.1mM **2** in 1:4 THF:mesitylene (v/v). A) 2D histogram, B) 1D histogram and C) Plateau length histogram adjusted for snap-back.[S10] All accumulated from 96% of 558 (total of 533) junctions. Red curves in B) and C) depict 2-peak Gaussian fits.

EC-STM-BJ Procedures and Data Analysis

Electrochemically controlled STM-BJ experiments were performed to measure the conductance of **1** in each of its three stable redox states: neutral state, radical cation and dication (see Table S1 below for potentials and results).

Experimental procedure

A flame-annealed Au disc was soaked overnight in a solution of 0.2 mM **1** in THF. This Au disc served as the STM substrate and an etched 0.25 mm Au wire, coated in polyethylene to minimise tip-substrate coupling, was used as the STM tip. A liquid cell was modified to allow two Pt electrodes to be inserted into 90 μL of $HMImPF_6$ ionic liquid electrolyte covering the Au disc substrate. The Pt electrodes were used as counter and reference electrodes. For all experiments the substrate-tip bias was constant at 100 mV and the sample potential between the substrate and the reference electrode was controlled with a bipotentiostat. Therefore, the sample potential was between the substrate and the reference electrode, while the tip was held at a potential 100 mV higher than the sample potential at all times.

After the STM tip was approached to the substrate, it was withdrawn by 4 nm and a CV was measured (scan rate: 100 mV s^{-1}). Withdrawal and CV measurement was repeated before each complete set of data was acquired at each sample potential. The stability of the ionic liquid meant that the location of the redox peaks in the CV never moved by more than about 50 mV. This meant it was possible to confidently measure the conductance of each redox state of **1**, but not intermediate states, e.g. at potentials corresponding to the peaks of oxidation or reduction waves. After recording a CV, the sample potential was swept from negative to positive until the sample potential corresponding to the desired state of the molecule was reached, at which point the sample potential was held fixed during collection of the EC-STM-BJ data set.

The focus was on three different sample potentials corresponding to three different states of **1**: neutral state, radical cation and dication. Fig. S11A shows the 1D histogram of

conductance traces acquired while **1** was in the neutral state. Two histogram peaks were apparent, which were interpreted in the same way as the STM-BJ results without electrochemical control. Fig. S11B shows the 1D histogram of conductance traces for the radical cation state. Two clear peaks were not apparent, but as a small shoulder appeared to be present the 1D histogram was fit to a 2-peak Gaussian for further analysis to account for any ‘half-length’ binding. Fig. S11C shows the 1D histogram of conductance traces for the dication state. Once again, although two clear peaks were not apparent, the 1D histogram was fit to a 2-peak Gaussian for further analysis.

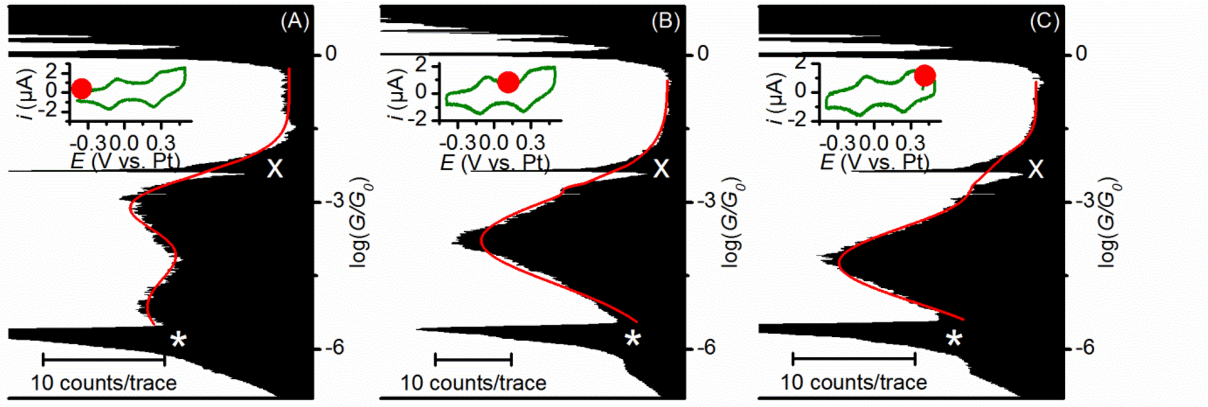

Fig. S11. 1D conductance histograms (each from ~1600 traces) from EC-STM-BJ experiments for A) neutral, B) radical cation and C) dication states, with inset CV acquired immediately before each measurement. In each case the red curve is a 2-peak Gaussian fit and the red circle on the inset CV marks the sample potential of the measurment. As in the main text, the white “x” marks an amplifier artefact which was masked for purposes of Gaussian fitting and the white “*” marks the peak from open-circuit noise.

Analysis

The 1D histogram for the neutral state (Fig. S11A) qualitatively matched the results from the STM-BJ results in measured in 1:4 THF:mesitylene (v/v). After fitting the data to a two-peak Gaussian model, the peak at ca. $10^{-3.0}$ G_0 also appeared to be present, albeit less prominently, in the 1D histograms for the other two measured states of **1** (Figs S11B and C). The interpretation of this peak must then be the same: the peak at ca. $10^{-3.0}$ G_0 is from a meta-stable conformation in the break junction which existed after the Au-Au junction broke,

but before the junction fully elongated with the molecule stretched between the Au electrodes. Again, this fits the proposed “half-length” conformation with a conductance pathway between the S atoms in the central BPTTF core of **1** and one of the terminal S atoms (see main text and Fig. S12). The conductance of this feature has only a weak potential dependence, supporting this hypothesis as the “half-length” pathway does not require conductance through the electroactive BPTTF core.

Unlike the peak at $10^{-3.0}$ G_0, the second, lower conductance peak (attributed to a “full-length” junction) did show a strong potential dependence. This is attributed to the differing extent of conjugation in the three redox states of the BPTTF unit. Table S1 summarises the conductance values measured in this study and plotted in Fig. 3D in the main text.

Table S1. Molecular conductances obtained in the EC-STM-BJ study

Electrochemical potential (/ V vs. Pt)	State	High conductance ($\log[G/G_0]$)^a	Low conductance ($\log[G/G_0]$)^a
–0.4	Neutral	–3.005±0.012	–5.17±0.03
+0.1	Radical cation	–2.70±0.02	–3.781±0.006
+0.4	Dication	–2.411±0.018	–4.239±0.004

^{a} error is uncertainty in the least-squares fit)

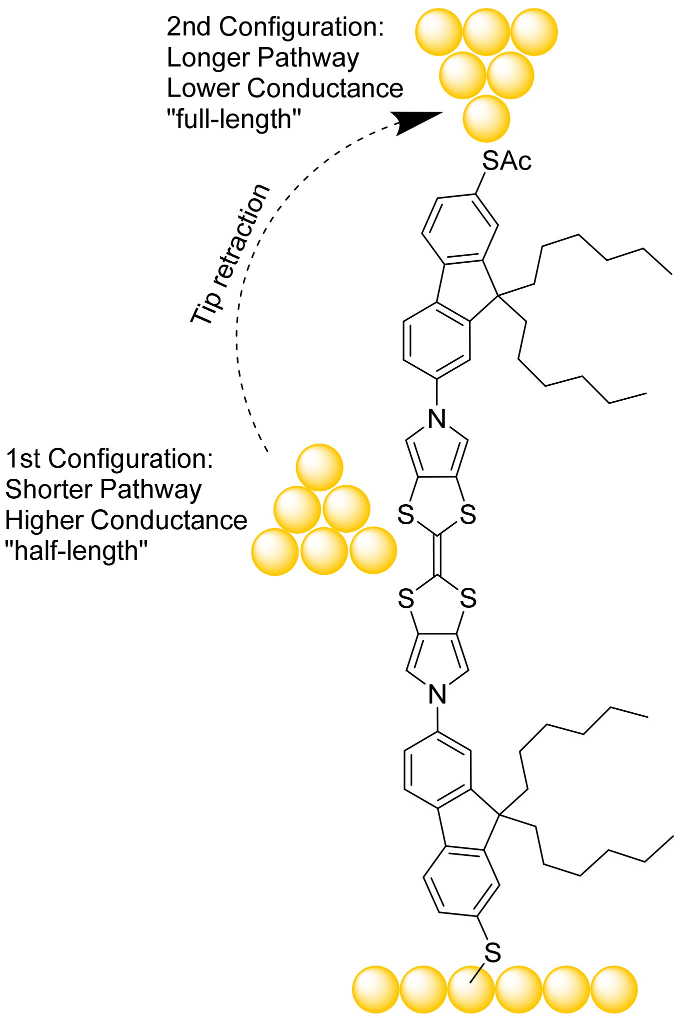

Fig. S12. Illustration of the two proposed binding configurations of the BPTTF wire **1** used to explain the conductance peaks observed in STM-BJ studies.

Computational Studies:

Junction Geometries

Computational Method

The ground state geometry of the isolated molecule **1** was first calculated using the density functional code SIESTA.[S11] A double-ζ plus polarisation (DZP) basis set was used along with norm conserving pseudopotentials and the exchange correlational functional was defined by the generalised gradient approximation (GGA).[S12] The energy cut-off which determines the spacing of the real space grid was defined as 150 Rydbergs and the molecule was relaxed until all forces on the atoms were less than 0.01 V/Å.

Gold electrodes were chosen, consisting of six layers of (111) gold terminated by a pyramid of gold atoms (which aim to model the nature of a junction formed in a break-junction measurement) and the optimum binding of the molecule between these electrodes was then calculated for two geometries. The first case is where the molecule is attached through both thiol groups ("full-length") and the second case is where the molecule is bound via one thiol and the second lead is attached via the central BPTTF unit ("half-length"). To calculate the binding energy we use the counterpoise method, which removes basis set superposition errors (BSSE). The binding distance d is defined as the distance between the tip of the gold pyramid and the sulfur atom of the thiol group. Here, molecule **1** is defined as entity A and the gold electrode as entity B. The ground state energy of the total system was calculated using SIESTA and is denoted $\mathrm{E}_{\mathrm{AB}}^{\mathrm{AB}}$, with the DFT parameters defined previously. The energy of each entity was then calculated in a fixed basis, which was achieved through the use of ghost atoms in SIESTA. Hence, the energy of the individual **1** molecule in the presence of the fixed basis is defined as $\mathrm{E}_{\mathrm{A}}^{\mathrm{AB}}$ and for the gold as $\mathrm{E}_{\mathrm{B}}^{\mathrm{AB}}$. The binding energy was then calculated using the following equation:

$$\text{Binding Energy} = E_{AB}^{AB} - E_{A}^{AB} - E_{B}^{AB} \qquad \text{(S1)}$$

Fig. S13 shows the binding energy versus distance for coupling to the thiol, which has a minimum at d = 2.4 Å and a value of 0.88 eV. In the case of calculations for the “full-length” molecule we use this contact geometry for each electrode.

For the second binding geometry (“half-length”), we calculated the binding of an electrode to the central BPTTF unit. Here we calculated the binding energy for 350 binding locations scanning along the axis between the two nitrogen atoms, varying the distance between the gold tip and the molecule between 2 and 3 Å. The result of this scan can be seen in Fig. S14. We find that the optimum binding location occurs for the tip directly above the sulfur atom as shown in Fig. S14 (left), with a distance of 2.55 Å. The binding energy for this contact is 0.35 eV which is significantly weaker than the terminal contact, but strong enough to suggest that such bindings will occur in the formation of molecular junctions.

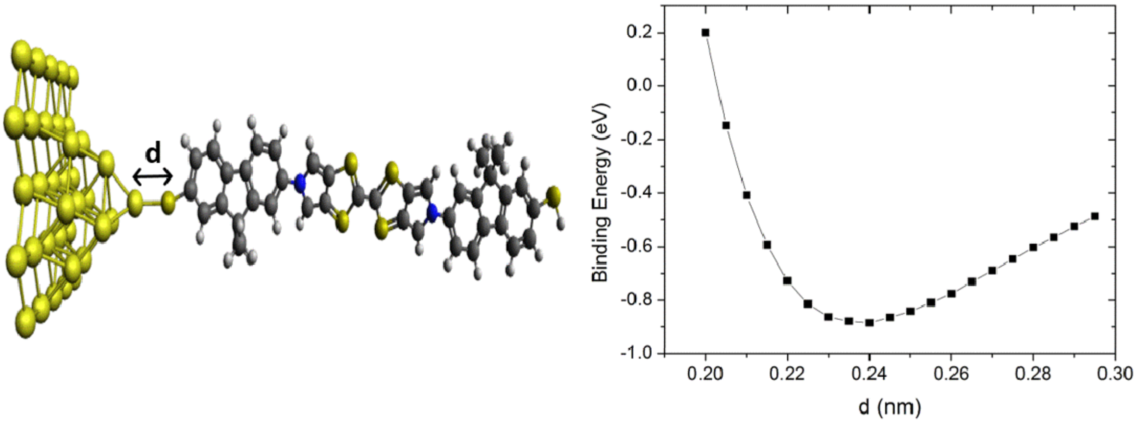

Fig. S13. (Left) Binding distance *d* between terminal sulphur atom and tip of gold electrode. (Right) Binding energy between sulphur atom and gold electrode as a function of distance d.

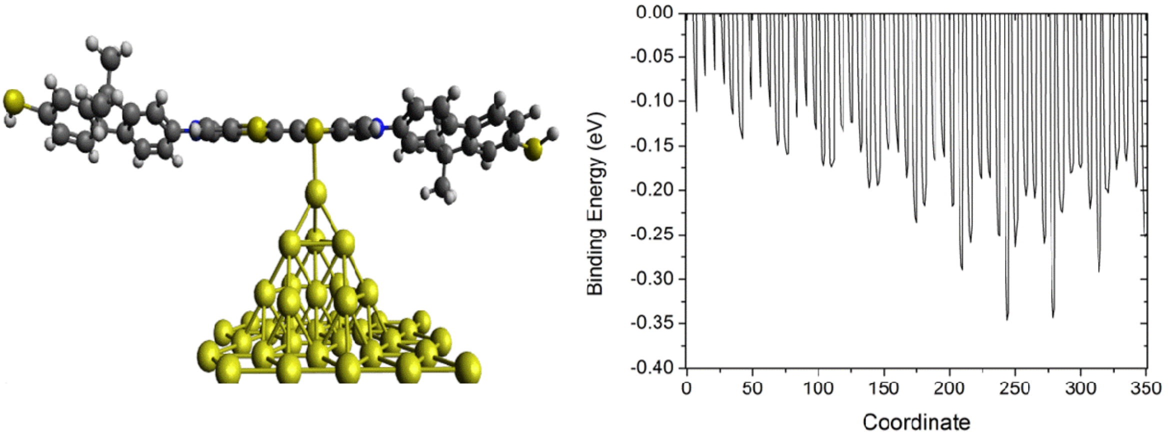

Fig. S14. (Left) Optimum binding geometry for binding to central BPTTF core. (Right) Binding energy to the central BPTTF core for 350 contact geometries.

To calculate the zero bias coefficient T(*E*) for each of these molecular junctions, in which the orientation of the gold leads can be arbitrary, we use a super cell approach. The structure, which we determine as the extended molecule, is isolated in the unit cell so that there are no interactions with neighbouring cells (i.e. there are vacuum buffer regions). The gold leads are constructed so that each layer consists of 25 gold atoms and the left and right electrodes each contain 9 layers. A Hamiltonian describing this structure is then extracted using SIESTA. Due to the terminating layers being artificial surfaces, these are removed and the sixth layer is treated as the principal layer of the electrode, with its Hamiltonian describing the bulk properties of the semi-infinite gold electrodes. For this number of layers the transmission coefficient T(*E*) has converged and further increasing the number of layers has no effect. The Fermi energy is defined as the Fermi energy of the extended molecule, which approaches that of the gold electrodes provided the number of layers of gold is large enough.

Reducing the size of the molecule

Molecule **1** has four hexyl chains attached to the fluorene sections of the molecule. Previous experimental work[S13] has shown that solubilising chains such as these play no role in the conductance of a molecule and can therefore be removed in theoretical calculations. In this work it is advantageous to remove them, as not only does it decrease the size of the system

to be studied, it also enables the charge-double layer to remain close to the molecular backbone in the charge variation study described below. To test that the removal of these groups has no effect we replaced the hexyl (C_6H_{13}) groups with methyl groups (CH_3); the resulting molecule is referred to as **1**-CH_3. The transmission coefficient T(*E*) was then calculated with gold electrodes attached to the thiol group in the optimum configuration described above. As shown in Fig. S15, molecule **1** (black line) and molecule **1**-CH_3 (red line) show identical transmission curves which enables this reduction of the molecule in all subsequent calculations.

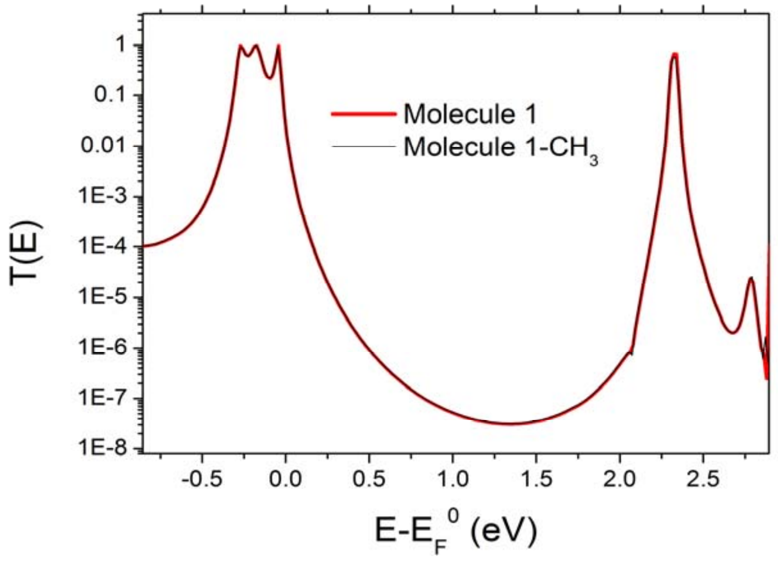

Fig. S15. Zero-bias transmission co-efficient T(*E*) versus electron energy *E* for molecules **1** and **1**-CH_3

Varying Molecular Charge

To model the electrochemical gating of the molecular wire within the junction we used a previously described method[S14] for controlling the amount of charge on the molecule between the gold electrodes. This utilises a charge double layer surrounding the wire, which can be moved a distance *d* (Fig. S16) from the molecular axis to control the amount of charge on the molecule; we determine the amount of charge on the molecule using a Mulliken population analysis. Here, we use a charge double layer of Na^+ and Cl^- ions and control the number of electrons moving between the molecule and gold so that the molecule

becomes positively charged and reaches the cation and dication states. The main difficulty in utilising this model is the applicability of this approach when the molecule is terminated by thiol anchor groups. When a thiol anchor group binds to gold electrodes the hydrogen atom is desorbed from the sulfur atom and a gold-sulfur bond is formed. This makes it difficult to determine the neutral charge as it differs from the case when the molecule is not in the junction.

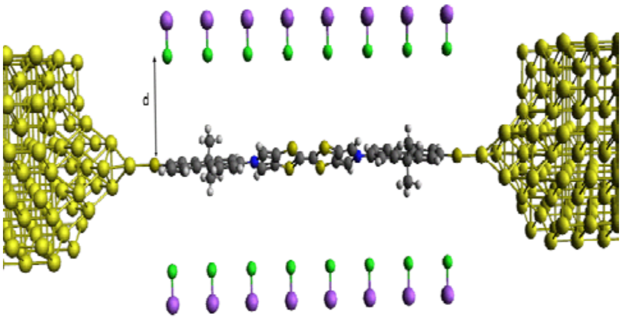

Fig. S16. Charge double layer a distance *d* from the molecule axis, with the negative side closest to the molecular backbone.

To overcome this we use the experimental data to fit the neutral state charge on the molecule. The measured conductance for molecule **1** under chemical potential control for the neutral state (N = 0) is $10^{-5.2}$ G_0. The charge double layer can be shifted by varying d (Fig. S16) so that the calculated conductance matches this value. When the number of electrons on molecule **1** = 235.05, the conductance $G(E–E_F^0$ = 0 eV) = $10^{-5.2}$ G_0. We use this to represent the neutral state (N = 0). The transmission coefficients for up spin and down spin electrons for N = 0 are shown in Fig. S17A and are identical, so in this neutral state the system is not spin-polarised.

The charge double layer is then moved closer to the molecule by decreasing the value of d until the N = –1 state is reached (i.e. the number of electrons on the molecule = 234.05). Fig. S17B shows that the HOMO resonance is now shifted closer to the Fermi energy and the up spin and down spin resonances are split. The dication state (N = –2) is reached by a further decrease in d and leads to a larger splitting of the HOMO resonances (Fig. S17C). The

conductance values calculated at the Fermi Energy (0 eV) can be seen in Fig. S17D and qualitatively match the experimental trends.

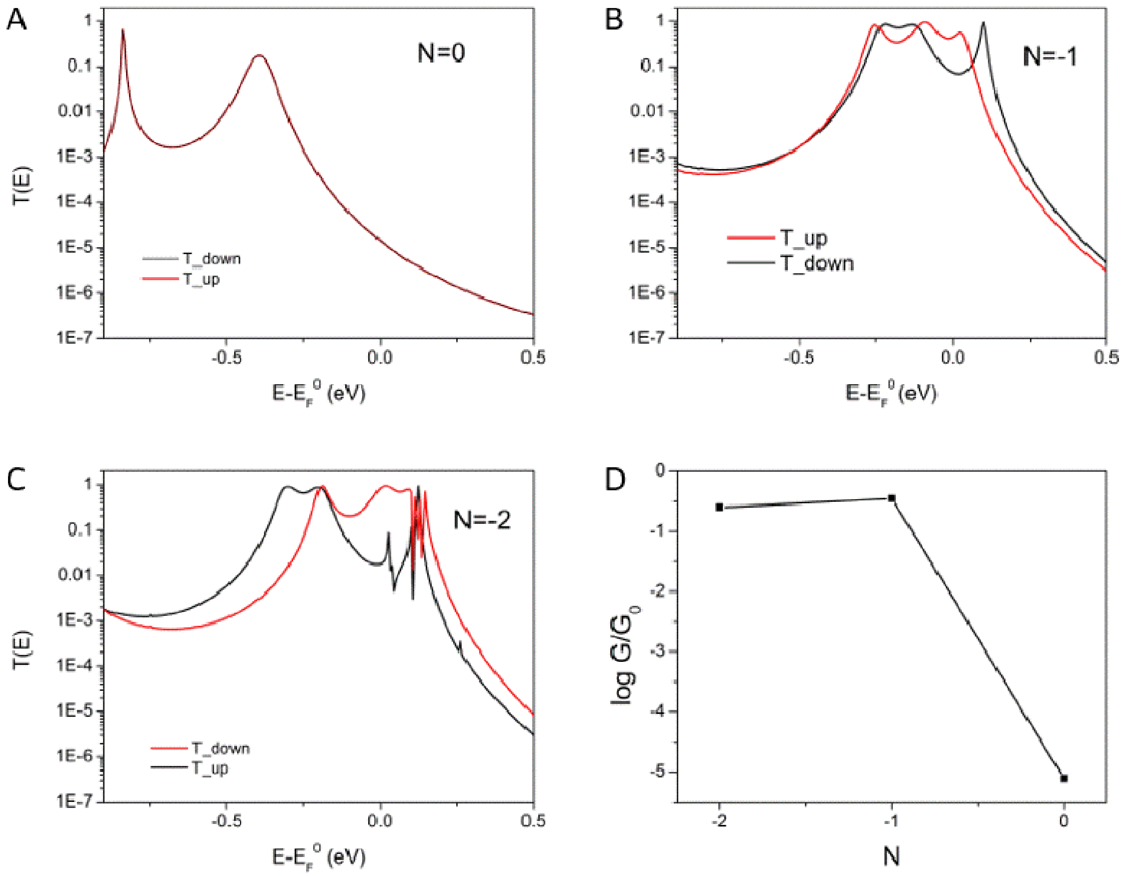

Fig. S17. Spin dependent zero-bias transmission coefficient T(E) for the (A) neutral (N = 0), (B) cation radical (N = –1) and (C) dication (N = –2) states of molecule **1**. (D) Conductance at $E–E_F^0$ = 0 eV for the three charge states.

References:

S1. L. J. O'Driscoll, S. S. Andersen, M. V. Solano, D. Bendixen, M. Jensen, T. Duedal, J. Lycoops, C. van der Pol, R. E. Sørensen, K. R. Larsen, K. Myntman, C. Henriksen, S. W. Hansen and J. O. Jeppesen, *Beilstein J. Org. Chem.*, 2015, **11**, 1112-1122.

S2. J. O. Jeppesen, K. Takimiya, F. Jensen, T. Brimert, K. Nielsen, N. Thorup and J. Becher, *J. Org. Chem.*, 2000, **65**, 5794-5805.

S3. Y.-Q. Huang, Q.-L. Fan, G.-W. Zhang, Y. Chen, X.-M. Lu and W. Huang, *Polymer*, 2006, **47**, 5233-5238.

S4. M. S. Maji, T. Pfeifer and A. Studer, *Chem. Eur. J.*, 2010, **16**, 5872-5875.

S5. S. H. Lee, T. Nakamura and T. Tsutsui, *Org. Lett.*, 2001, **3**, 2005-2007.

S6. B. Xu and N. J. Tao, *Science*, 2003, **301**, 1221-1223.

S7. C. Huang, A. V. Rudnev, W. Hong and T. Wandlowski, *Chem. Soc. Rev.*, 2015, **44**, 889-901.

S8. L. Xiang, T. Hines, J. L. Palma, X. Lu, V. Mujica, M. A. Ratner, G. Zhou and N. Tao, *J. Am. Chem. Soc.*, 2016, **138**, 679-687.

S9. Y. Komoto, S. Fujii, K. Hara and M. Kiguchi, *J. Phys. Chem. C*, 2013, **117**, 24277-24282.

S10. W. Hong, D. Z. Manrique, P. Moreno-García, M. Gulcur, A. Mishchenko, C. J. Lambert, M. R. Bryce and T. Wandlowski, *J. Am. Chem. Soc.*, 2012, **134**, 2292-2304.

S11. M. S. José, A. Emilio, D. G. Julian, G. Alberto, J. Javier, O. Pablo and S.-P. Daniel, *J. Phys.: Condens. Matter*, 2002, **14**, 2745-2779.

S12. J. P. Perdew, K. Burke and M. Ernzerhof, *Phys. Rev. Lett.*, 1996, **77**, 3865-3868.

S13. R. Huber, M. T. González, S. Wu, M. Langer, S. Grunder, V. Horhoiu, M. Mayor, M. R. Bryce, C. Wang, R. Jitchati, C. Schönenberger and M. Calame, *J. Am. Chem. Soc.*, 2008, **130**, 1080-1084.

S14. Y. Li, M. Baghernejad, A.-G. Qusiy, D. Zsolt Manrique, G. Zhang, J. Hamill, Y. Fu, P. Broekmann, W. Hong, T. Wandlowski, D. Zhang and C. Lambert, *Angew. Chem. Int. Ed.*, 2015, **54**, 13586-13589.
